# Mitochondrial outer membrane protein MTUS1/ATIP1 exerts antitumor effects through ROS-induced mitochondrial pyroptosis in head and neck squamous cell carcinoma

**DOI:** 10.7150/ijbs.94795

**Published:** 2024-04-22

**Authors:** Dongxiao Tang, Luodan Zhao, Shuojin Huang, Wuguo Li, Qianting He, Anxun Wang

**Affiliations:** 1 Department of Oral and Maxillofacial Surgery, The First Affiliated Hospital, Sun Yat-Sen University, Guangzhou, Guangdong 510080, China.; 2 Department of Stomatology, The Third Affiliated Hospital, Sun Yat-Sen University, Guangzhou, Guangdong 510630, China.; 3 Department of Stomatology, Sun Yat-Sen Memorial Hospital, Sun Yat-Sen University, Guangzhou, Guangdong 510120, China.; 4 Animal Experiment Center, The First Affiliated Hospital, Sun Yat-Sen University, Guangzhou, Guangdong 510080, China.

**Keywords:** MTUS1/ATIP1, mitochondria, HNSCC, ROS, pyroptosis

## Abstract

We showed that microtubule-associated tumor suppressor gene (MTUS1/ATIP) downregulation correlated with poor survival in head and neck squamous cell carcinoma (HNSCC) patients and that MTUS1/ATIP1 was the most abundant isoform in HNSCC tissue. However, the location and function of MTUS1/ATIP1 have remain unclear. In this study, we confirmed that MTUS1/ATIP1 inhibited proliferation, growth and metastasis in HNSCC in cell- and patient-derived xenograft models *in vitro* and *in vivo*. MTUS1/ATIP1 localized in the outer mitochondrial membrane, influence the morphology, movement and metabolism of mitochondria and stimulated oxidative stress in HNSCC cells by directly interacting with MFN2. MTUS1/ATIP1 activated ROS, recruiting Bax to mitochondria, facilitating cytochrome c release to the cytosol to activate caspase-3, and inducing GSDME-dependent pyroptotic death in HNSCC cells. Our findings showed that MTUS1/ATIP1 localized in the outer mitochondrial membrane in HNSCC cells and mediated anticancer effects through ROS-induced pyroptosis, which may provide a novel therapeutic strategy for HNSCC treatment.

## Introduction

Head and neck squamous cell carcinoma (HNSCC) is the most common head and neck cancer 1,2. Till now, the overall 5-year survival of HNSCC has not improved significantly beyond 50% 3. Recently, major breakthroughs in immunotherapy and targeted therapy have brought substantially more effective treatment strategies to malignant tumor patients 4-6. However, few target drugs have been found that are effective for HNSCC 7. Therefore, there is an urgent need to identify more effective and safe therapeutic targets for HNSCC. Microtubule-associated tumor suppressor gene (MTUS1) is an 8p22 candidate tumor suppressor gene encoding a family of angiotensin II (AT2) receptor-interacting proteins (ATIPs) such as ATIP1, ATIP2, ATIP3a, ATIP3b and ATIP4^8^-^10^. The downregulation of MTUS1 is implicated in multiple types of cancers 11,12. Our previous studies reported that MTUS1 transcript variant 5 (ATIP1) was the most abundant isoform in HNSCC tissues 13. The downregulation of MTUS1/ATIP1 in HNSCC is correlated with poor overall survival and ATIP1 restoration expression led to apoptosis and a reduction in cell proliferation in HNSCC cells^14^. Thus, MTUS1/ATIP1 may be a new treatment target for HNSCC.

Cancer cells expend increased energy to maintain a high rate of cell division, which requires more mitochondria and highly active mitochondrial metabolism 15. However, mitochondrial metabolism produces high level of reactive oxygen species (ROS), which are associated with cancer development and cancer cell death. Excessive levels of ROS enhance cellular oxidative stress, which damages DNA, proteins and lipids, leading to apoptotic or necroptotic cell death 16. Many researches had revealed that mitochondrial outer membrane permeabilization (MOMP) driven by proapoptotic members of the B-cell lymphoma 2 (BCL2) family (mainly BAX and BAK) cell death 17, including apoptosis, necroptosis, pyroptosis and ferroptosis 18,19. Pyroptosis is characterized by cell swelling, large pores in the plasma membrane, cell lysis and chromatin fragmentation, resulting in the release of cell contents and proinflammatory molecules and playing an important role in cancers 20,21. Zhang *et al.* showed that pyroptosis suppressed tumor growth in melanoma and triple-negative breast cancer 22. Chemotherapy drugs such as lobaplatin 23, doxorubicin 24 and cisplatin 25 was also found to kill cancer cells by inducing pyroptosis.

Till now, whether the location of MTUS1/ATIP in mitochondria had not been fully clarify. Molina el al. had revealed that ATIP3 is localized with microtubules and involved in the delay of mitosis and restriction of cell migration 26. Rodrigues-Ferreira *et al.* also found that ATIP4 possesses a transmembrane domain and localizes to plasma membrane 27. But the location of ATIP1 had not been fully clarify. To investigate whether the localization of MTUS1/ATIP1 in mitochondria and how this localization influences mitochondrial function in HNSCC, we employed the methods of Immunofluorescence and coimmunoprecipitation, Protease K assays, MitoTracker staining, seahorse assays, etc. Pyroptosis and its mechanism were examined by high-content microscopic imaging, immunofluorescence staining and western blotting and lactate dehydrogenase (LDH) release assay. Finally, the anticancer effect of MTUS1/ATIP1 was confirmed in HNSCC cell-derived xenograft (CDX) and patient-derived xenograft (PDX) models. In this study, we found that MTUS1/ATIP1 was localized in the outer mitochondrial membrane and exerted anticancer effects on HNSCC through ROS-induced pyroptosis; thus, MTUS1/ATIP1 may be a novel, promising therapeutic target for the clinical treatment of HNSCC.

## Materials and methods

### Cell Culture and transfection

The HNSCC cell lines SCC9, SCC15 and SCC25 were purchased from ATCC and HSC3 was purchased from the cell bank of the Japanese Collection of Research Bioresource (CRB), with the cell line STR authentication report. SCC9, SCC15 and SCC25 cells were cultured in DMEM/F12 (Gibco, Carlsbad, CA) supplemented with 10% fetal bovine serum (FBS; Gibco, Carlsbad, CA) and 1% penicillin/streptomycin (P/S; Gibco, Carlsbad, CA) and HSC3 cells were cultured in DMEM (Gibco, Carlsbad, CA) supplemented with 10% FBS and 1% P/S. The cells were cultured in a 5% CO2 incubator at 37°C.

Lentiviral vectors encoding MTUS1/ATIP1 shRNA, MFN2 shRNA, BAX shRNA or scramble shRNA were purchased from Genechem (Shanghai, China). Three shRNA sequences targeting human MTUS1/ATIP1, MFN2 or BAX were tested, and the sequence with the best knockdown effect was chosen for further study. Lentiviral vectors containing MTUS1 (ATIP1) cDNA (ID: NM_020749.4) were purchased from iGene Biotechnology (Guangzhou, China). HNSCC cells were infected with lentiviruses at a multiplicity of infection (MOI) of 10 or 25. The detailed sequences of the MTUS1 (ATIP1), MFN2 or BAX shRNA are listed in Table S1.

### Animals study

BALB/c nude mice were purchased and fed in a specific pathogen-free (SPF) environment in the Animal Experiment Center of the First Affiliated Hospital of Sun Yat-sen University (Guangzhou, China). The Ethics Committee of the First Affiliated Hospital of Sun Yat-Sen University approved the animal experiments and the use of tissue samples from HNSCC patients who provided informed consent ([2022]229).

The **CDX model** was established as previously described 28-30. 6-week-old female BALB/c mice were injected with luciferase-labelled Lv-OE-ATIP1 or Lv-shATIP1 infected SCC9 cells into the right flanks [1×10^6^ cells in 100 μL vehicle (1:1 mixture of DMEM and Matrigel matrix)] for subcutaneous tumorigenesis model, the right lateral tongue (1×10^5^ cells in 20 μL vehicle) for orthotopic xenograft tongue cancer model or the right hind footpads (1×10^5^ cells in 50 μL DMEM) for lymph node metastasis model, respectively. The xenograft sizes were measured every 3 days and tumor volumes were calculated as follows: tumor volume (mm^3^) = ½ × length × width^2^. Bioluminescence imaging was performed using Xenogen IVIS Spectrum imaging system (Xenogen, Alameda, CA) 10 minutes after the mice were i.p. injected with 150 mg/kg D-luciferin. The images were quantified as photons/s/cm^2^/sr. The mice were euthanized 28 days after subcutaneous or tongue injection, and mice subjected to hind footpad injection were euthanized 6 weeks after injection. After sacrifice, tumors were extracted, weighed, measured and then embedded, sectioned and stained for H&E staining. The popliteal lymph nodes of the right hind limbs were counted and analyzed by H&E staining.

The **PDX models** were established as previously described 31. In brief, fresh tumor specimens from two patients were collected during surgery, dissected into pieces and subcutaneously implanted into the flanks of 4- to 6-week-old female BALB/c nude mice Table S2. Third-generation PDX models were treated by intratumoral injection of Lv-OE-ATIP1 once per week as previously described 32,33. Tumor volume and body weight were measured every 3 days. The mice were sacrificed on day 28, and the xenograft tissues were embedded in paraffin for H&E staining. The TGI rate was calculated as follows: TGI rate (%) =(1-Tn/Cn) ×100%. Tn and Cn indicate the relative tumor volumes on day n in the treated or control groups.

### Cell proliferation assay, colony formation assays and wound healing assay

Cell proliferation was measured using Cell Counting Kit-8 assays (CCK-8) (Dojindo, Kumamoto, Japan) according to the manufacturer's instructions. In brief, the indicated cells were plated at 3×10^3^ cells per well in 96-well plates and cultured for the indicated time. CCK-8 solution was added to the cell culture medium. After 2 h, the absorbance at 450 nm was recorded using a microplate reader (Bio-Tek, Winooski, VT).

For colony formation assays, the indicated cells were plated into six-well plates at a density of 200 cells/mL in triplicate and cultured for 14 days, after which the colonies were fixed with 4% formaldehyde and stained with 0.1% crystal violet. Then, the colonies were photographed and counted (larger than 1 mm, >50 cells/clone).

For wound healing assays, the indicated cells were seeded into 6-well culture plates and cultured in FBS-free medium. After the formation of a confluent cell monolayer, the monolayer was scratched with a sterile 200-μL pipette tip and cultured in fresh medium containing 2% FBS. The status of cell migration was observed at 0 hour and 24 hours in 3 random fields of view using Zeiss axio observer z1 microscope (Zeiss, Jena, Germany). The obtained images were analyzed by ImageJ software.

### Quantitative real-time PCR (qRT‒PCR), western blotting and coimmunoprecipitation

For qRT-PCR, total RNA was extracted with TRIzol reagent (Invitrogen, Carlsbad, CA). Reverse transcription was performed with the PrimeScript RT Master Mix kit (Takara, Kyoto, Japan), and quantitative PCR (qPCR) was carried out in a StepOnePlus Real-Time PCR Instrument (Thermo Fisher, Waltham, MA) using SYBR Green Real-time PCR Master Mix. Gene expression was normalized using 36B4 or GAPDH as the internal control, and relative expression was calculated using the ^ΔΔ^Ct method. All primer sequences are listed in Table S3.

For Western blotting, total cellular protein was harvested by living cells in ice-cold RIPA buffer (Sigma-Aldrich, Saint Louis, MO). Aliquots (30 μg) of cellular proteins were resolved via SDS-PAGE and electrotransferred onto polyvinylidene fluoride (PVDF) membranes. The membrane was blocked with 3% bovine serum albumin (BSA), probed with primary antibodies overnight at 4°C and then incubated with secondary antibodies. Then, the antibody-protein complexes were detected using enhanced chemiluminescence (ECL) reagents (Abcam, Cambridge, MA). All primary antibodies used are listed in Table S4.

For coimmunoprecipitation, cellular proteins were collected by using IP buffer (Thermo Fisher, Waltham, MA). The protein lysates were immunoprecipitated with primary antibodies or control IgG and a Dynabeads Coimmunoprecipitation Kit (Thermo Fisher, Waltham, MA) and then subjected to electrophoresis on an SDS-polyacrylamide gel for western blotting.

### Immunofluorescence staining and MitoTracker Red staining

For immunofluorescence staining, the indicated cells were seeded in confocal dishes and cultured overnight. Then, the cells were rinsed and fixed with 4% paraformaldehyde (PFA) followed by incubation with 0.5% Triton X-100 at room temperature for 15 min, blocked with 5% BSA for 1 h and incubated with primary antibodies overnight at 4°C, followed by incubation with appropriate secondary antibodies. Isotype IgGs for each specific antibody were used as negative controls. Nuclei were stained with DAPI (Thermo Fisher, Waltham, MA). To investigate the subcellular localization of MTUS1/ATIP1, HNSCC cells were infected with Lv-OE-ATIP1-Flag at an MOI of 10 for 6 h and immediately fixed with PFA to reduce mitochondrial collapse caused by MTUS1/ATIP1 overexpression as much as possible. All primary antibodies used are listed in Table S5.

To visualize mitochondria in live cells, the indicated cells were incubated with MitoTracker Red (Invitrogen, Carlsbad, CA) at 37°C for 40 min and rinsed with PBS. Time-lapse live-cell imaging analysis of mitochondria and confocal imaging were performed with Zeiss LSM880 confocal microscope (Zeiss, Jena, Germany) as previous described 34. The ratio of cells with collapsed mitochondria was quantified in 4 randomly chosen fields per dish.

### Subcellular protein extraction, mitochondria isolation and Protease K assay

Subcellular protein extraction and mitochondria isolation were performed using the subcellular protein fractionation kit (Thermo Fisher, Waltham, MA) and mitochondria isolation kit (Thermo Fisher, Waltham, MA), respectively, according to the manufacturer's instructions as previously reported 35,36. The fractions were subjected to SDS-PAGE and examined by Western blotting.

For the Protease K assay, the mitochondrial fraction was digested with different concentrations of Protease K (Sigma-Aldrich, Saint Louis, MO) for 5 min. Mitochondrial proteins such as MFN1, MFN2, TOMM20 (mitochondrial outer membrane protein) and Timm23 (mitochondrial inner membrane protein) were analyzed by Western blotting with the corresponding antibodies.

### Seahorse assay

The OCR and ECAR were measured using an Agilent Seahorse XF Cell Mito Stress Test Kit and Glycolysis Stress Test Kit (Agilent, Santa Clara, CA), respectively. Briefly, the indicated cells were plated in a 96-well XF cell culture microplate (Agilent, Santa Clara, CA). Cells were left to adhere for one hours at 37°C in the absence of CO_2_. OCR was then measured in basal conditions and upon the sequential addition of oligomycin, carbonyl cyanide-4-(trifluoromethoxy) phenylhydrazone (FCCP), and rotenone/antimycin A to determine maximum respiration and spare respiratory capacity of tested cells. ECAR was then measured in basal conditions and upon the sequential addition of glucose, oligomycin, and 2-deoxyglucose (2-DG), to determine the glycolytic rate and the glycolytic capacity of tested cells. The OCR and ECAR were calculated using the Seahorse XF Report Generator (Agilent, Santa Clara, CA).

### Mitochondrial membrane potential (MMP) assay and ATP assay

A JC-1 MMP measurement kit (Beyotime, Shanghai, China) was used to measure MMP (ΔΨm). Briefly, the indicated cells (2×10^5^) were resuspended in 0.5 mL of JC-1 working solution and incubated for 20 min. The cells were washed and analyzed by flow cytometry (Biosciences, Franklin Lakes, NJ).

A luciferase-based enhanced ATP assay kit (Beyotime, Shanghai, China) was used to determine ATP levels. Briefly, the indicated cells were lysed and centrifuged at 12,000× g for 5 min. The supernatant was added to a 96-well plate containing ATP detection working solution. Luminescence was detected by a multifunction microplate reader (Perkin Elmer, USA). The protein concentration of each group and an ATP standard curve were used to calibrate the ATP levels in the cells.

### ROS assay

ROS assay kits (Beyotime, Shanghai, China) was used to determine ROS levels. Briefly, the indicated cells were seeded in 6-well plates, collected and incubated in DCFH-DA for 40 min. The fluorescence intensity of DCF was measured using a CytoFLEX flow cytometer (Beckman Coulter, Brea, CA). ROS levels were determined according to a DCF-standard curve and are presented as pmol DCF formed/min/mg protein.

### High-content microscopic imaging

High-content microscopic imaging was used to observe the morphology of pyroptotic cells. Briefly, the indicated cells were plated in a 96-well plate and cultured overnight. Live cell images were taken using the Bio-Tek Lionheart FX automated high-content microscope (Bio-Tek, Winooski, VT) under normal cells culture condition. Images were obtained every 10 minutes for 24 hours. Image analysis was performed using Gen5 software (Bio-Tek, Winooski, VT).

### LDH release assay

The CytoTox 96 Non-Radioactive Cytotoxicity Assay Kit (Promega, Madison, WI) was used to detect cellular LDH release according to the manufacturer's instructions. The LDH release rates were calculated as previously described 35.

### Statistical analysis

The data were statistically analyzed with GraphPad Prism v9.3 (GraphPad Software, San Diego, CA) and SPSS 26.0 software (SPSS Inc, Chicago, IL). A two-tailed *t* test was used to analyze the differences between two groups, and one-way *ANOVA* was used for three or more groups. The data are presented as the mean±SD or SEM. Differences at *P*<0.05 were considered statistically significant.

## Results

### MTUS1/ATIP1 was localized in the outer mitochondrial membrane

**Figure 1:**
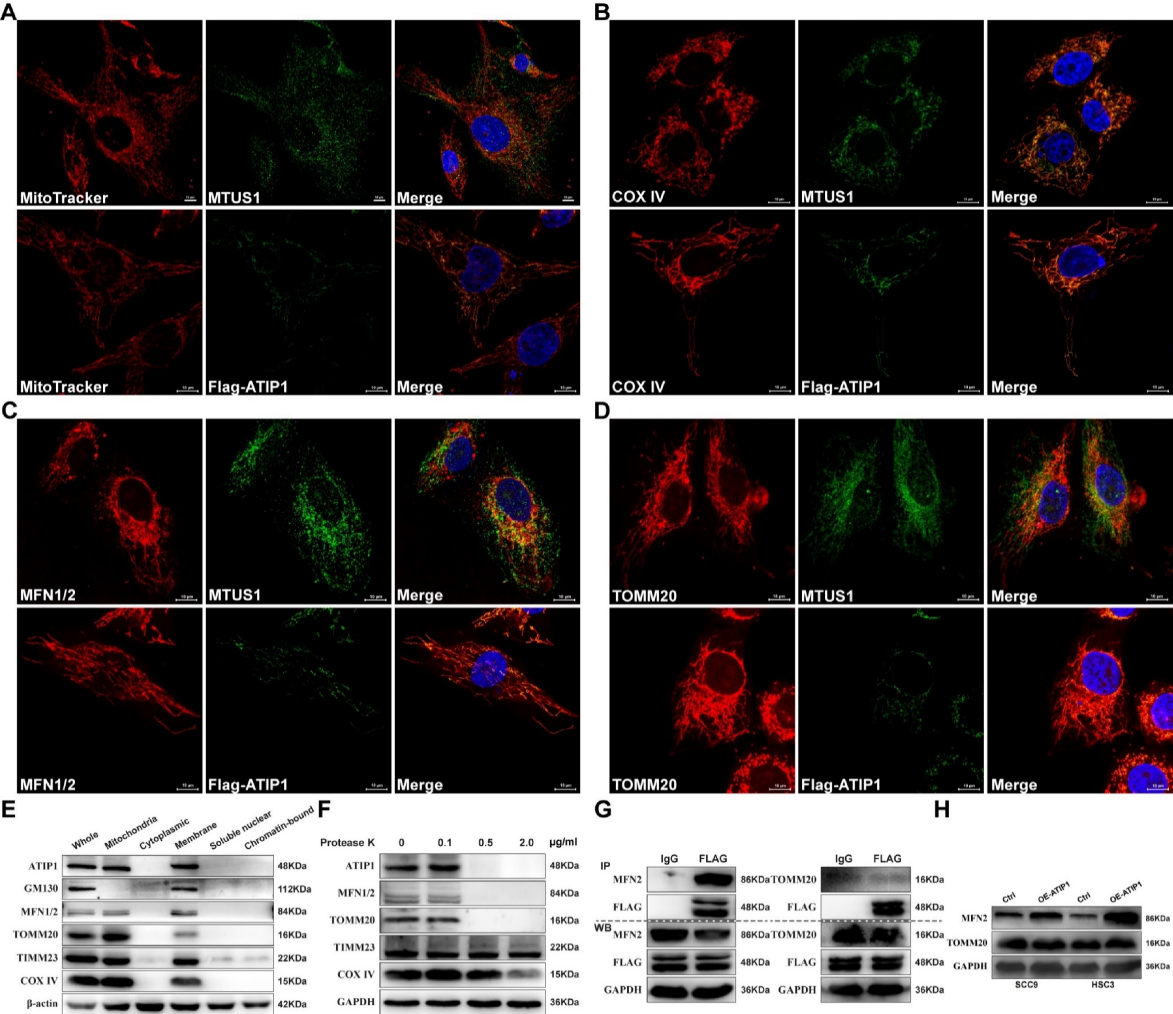
** MTUS1/ATIP1 was localized at the outer mitochondrial membrane in HNSCC cells.** (**A-D**) HNSCC cells were subjected to immunofluorescent staining with MTUS1/Flag (green) and MitoTracker red staining (A) or COX IV (red) (B) or MFN1/2 (red) (C) or TOMM20 (red) (D) and counterstained with DAPI (blue). (**E**) Equal amounts of whole cell lysate, mitochondria and cytoplasmic, membrane, soluble nuclear and chromatin-bound extractions from HNSCC cells were analyzed by western blotting using the indicated antibodies including mitochondria marker (COX IV and Timm23), MOM marker (MFN2 and TOMM20) and Golgi marker (GM130). (**F**) Mitochondria were isolated and incubated with protease K for 5 min and then subjected to western blotting to detect ATIP1, mitochondria marker (COX IV and Timm23) and MOM marker (MFN2 and TOMM20). (**G**) Cell lysates from HNSCC cells transduced with lentivirus containing ATIP1-Flag were subjected to immunoprecipitation with an antibody against Flag, followed by western blotting with the indicated antibodies (MFN2 or TOMM20). (**H**) Cell lysates were subjected to western blotting with the MFN2 or TOMM20 antibodies in MTUS1/ATIP1-overexpressed or control HNSCC cells. Scale bar, 10 mm. β-actin or GAPDH was used as loading proteins.

To determine whether the localization of MTUS1/ATIP1 in mitochondria, we first established the HNSCC cell lines with stable overexpression of FLAG-tagged MTUS1/ATIP1 Figure S1A). MTUS1/ATIP1, mitochondria and its related markers were examined by immunofluorescence or MitoTracker analysis, respectively. As shown in Figure 1A-D, endogenous or Flag-tagged MTUS1/ATIP1 was colocalized with mitochondria and also colocalized with the mitochondrial marker COX IV and the mitochondrial outer membrane (MOM) proteins MFN1/2 and TOMM20 in HNSCC cells. From the subcellular protein extracted, MTUS1/ATIP1 was highly enriched in the purified mitochondrial fraction, which was characterized by the MOM markers MFN1/2 and Tomm20 and the mitochondrial markers COX IV and TIMM23 (Figure 1E). Furthermore, when crude mitochondrial fractions were incubated with protease K, MTUS1/ATIP1 was disappeared with MFN1/2 and TOMM20, but not the other mitochondrial proteins (including COX IV and TIMM23) (Figure 1F).

To investigate the interaction between MTUS1/ATIP1 and MOM proteins (MFN2 and TOMM20), cell lysates from HNSCC cells transfected with FLAG-tagged MTUS1/ATIP1 were examined by coimmunoprecipitation with a FLAG antibody. As shown in Figure 1G, MFN2 but not TOMM20 was pulled down by the Flag antibody, which suggested that MTUS1/ATIP1 directly interacted with MFN2. Moreover, MTUS1/ATIP1 overexpression was accompanied by increased MFN2 but not TOMM20 expression in HNSCC cells (Figure 1H). These data indicated that MTUS1/ATIP1 localized in the outer mitochondrial membrane in HNSCC cells and interacted with the MFN2 protein.

### MTUS1/ATIP1 influenced mitochondrial function and metabolism in HNSCC cells

**Figure 2:**
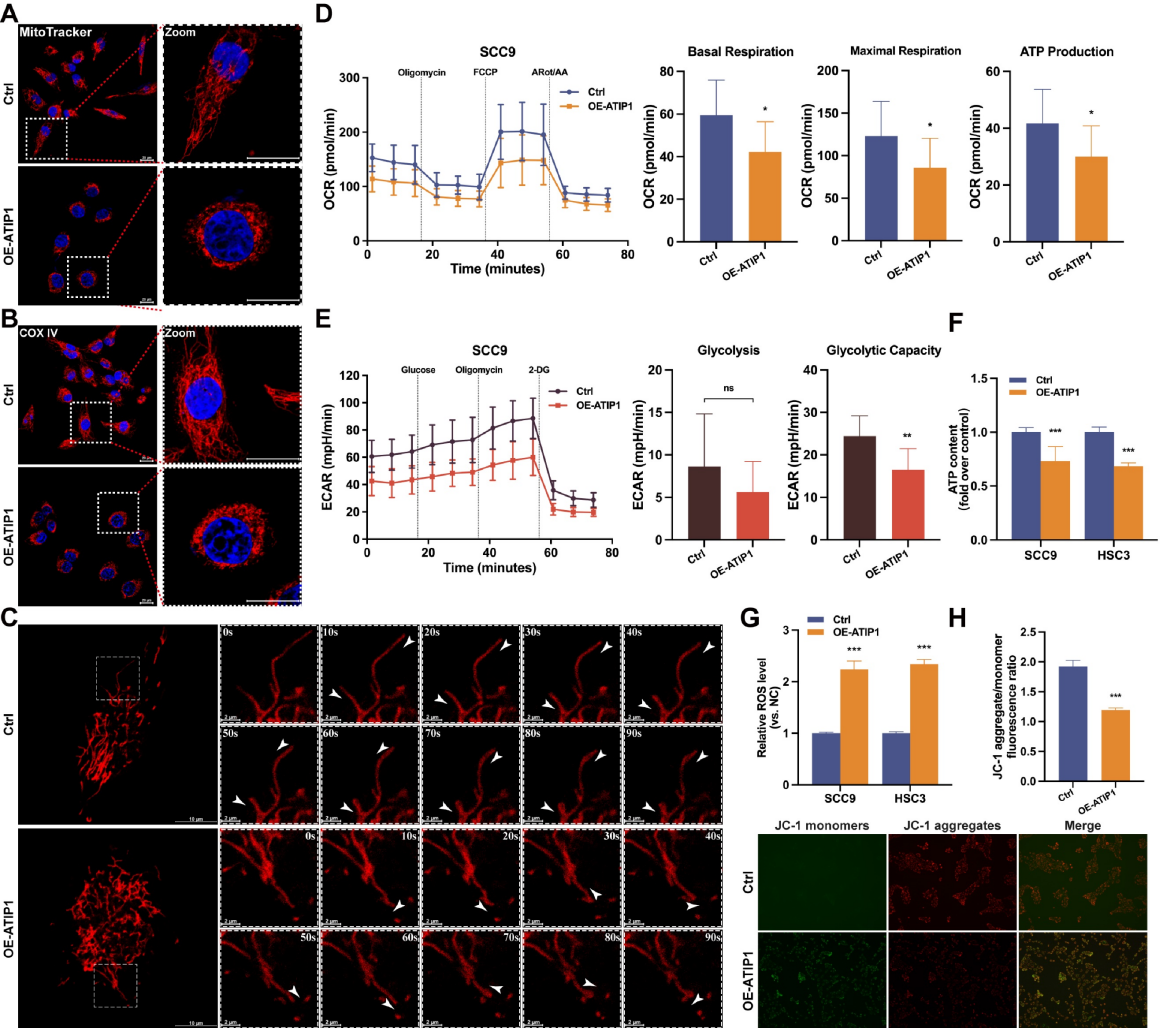
** MTUS1/ATIP1 influenced mitochondrial function and metabolism in HNSCC cells.** (**A-B**) MitoTracker Red staining (A) or immunofluorescence staining with COX IV (B) were used to detect the distribution of mitochondria under a confocal microscope in MTUS1/ATIP1-overexpressed or control HNSCC cells. Scale bars, 20 μm. (**C**) Mitochondrial real-time motions of HNSCC cells, stained by MitoTracker Red were detected under a confocal time-lapse live-cell analysis system. White arrowheads indicate direction of movement of single moving mitochondria. (**D-E**) MTUS1/ATIP1-overexpressed or control SCC9 cells was determined using oxygen consumption rate (OCR) and extracellular acidification rate (ECAR) assays. (**F**) Intracellular ATP levels were measured by a bioluminescence ATP determination assay in MTUS1/ATIP1-overexpressed or control HNSCC cells. (**G**) MTUS1/ATIP1-overexpressed or control HNSCC cells were stained with DCFH-DA and then ROS levels were detected using flow cytometry. (**H**) The MMP was detected by a fluorescence microscope using JC-1 probe staining in MTUS1/ATIP1-overexpressed or control HNSCC cells. All data are presented as the mean ± SEM of three independent experiments. ns, not statistically significant (*P*≥ 0.05); *P < 0.05; **P < 0.01; ***P < 0.001.

First, the effect of MTUS1/ATIP1 on mitochondrial morphology in HNSCC cells was examined. As shown in Figure 2A and S2A, long tubular mitochondria were spread out in a highly interconnected network in the control group, while collapse of mitochondria were tightly packed around the nuclei in MTUS1/ATIP1-overexpression group. Immunofluorescence staining of the mitochondrial marker COX IV further confirmed the presence of perinuclear mitochondrial collapse in MTUS1/ATIP1-overexpressed cells (Figure 2B and S2B). Then mitochondrial movement in living HNSCC cells was observed under confocal microscopy. In control group, the majority of moving mitochondria traveled in a directed manner, while the mitochondrial motility was random (Brownian motion) and directed motion was decreased in MTUS1/ATIP1-overexpressed HNSCC cells (Figure 2C and Video S1-S2).

To determine how MTUS1/ATIP1 affects mitochondrial metabolism, Seahorse assays were performed. The OCR results showed a substantial reduction in basal respiration, maximal respiration and ATP production in MTUS1/ATIP1-overexpressed HNSCC cells, which indicates the inhibition of mitochondrial oxidative phosphorylation (Figure 2D and S2C). The ECAR results showed no overt differences in basal glycolysis, but a significant reduction was observed in oligomycin-induced glycolysis, suggesting a defect in glycolytic metabolism (Figure 2E and S2D). We also established the HNSCC cell lines with stable knockdown MTUS1/ATIP1 (Figure S1B) and found that ATP levels were significantly reduced in MTUS1/ATIP1-overexpressed HNSCC cells and increased in MTUS1/ATIP1-knockdown HNSCC cells (Figure 2F and S2E). Moreover, MTUS1/ATIP1 overexpression significantly increased intracellular ROS levels (Figure 2G and S2F) and decreased the mitochondrial membrane potential (MMP), shown as decreased in the red/green fluorescence intensity ratio (Figure 2H). These above results suggested that MTUS1/ATIP1 influenced mitochondrial function and metabolism in HNSCC cells, altered the mitochondrial distribution and movement and stimulated oxidative stress.

### MTUS1/ATIP1 induced ROS-dependent mitochondrial pyroptosis in HNSCC cells

**Figure 3:**
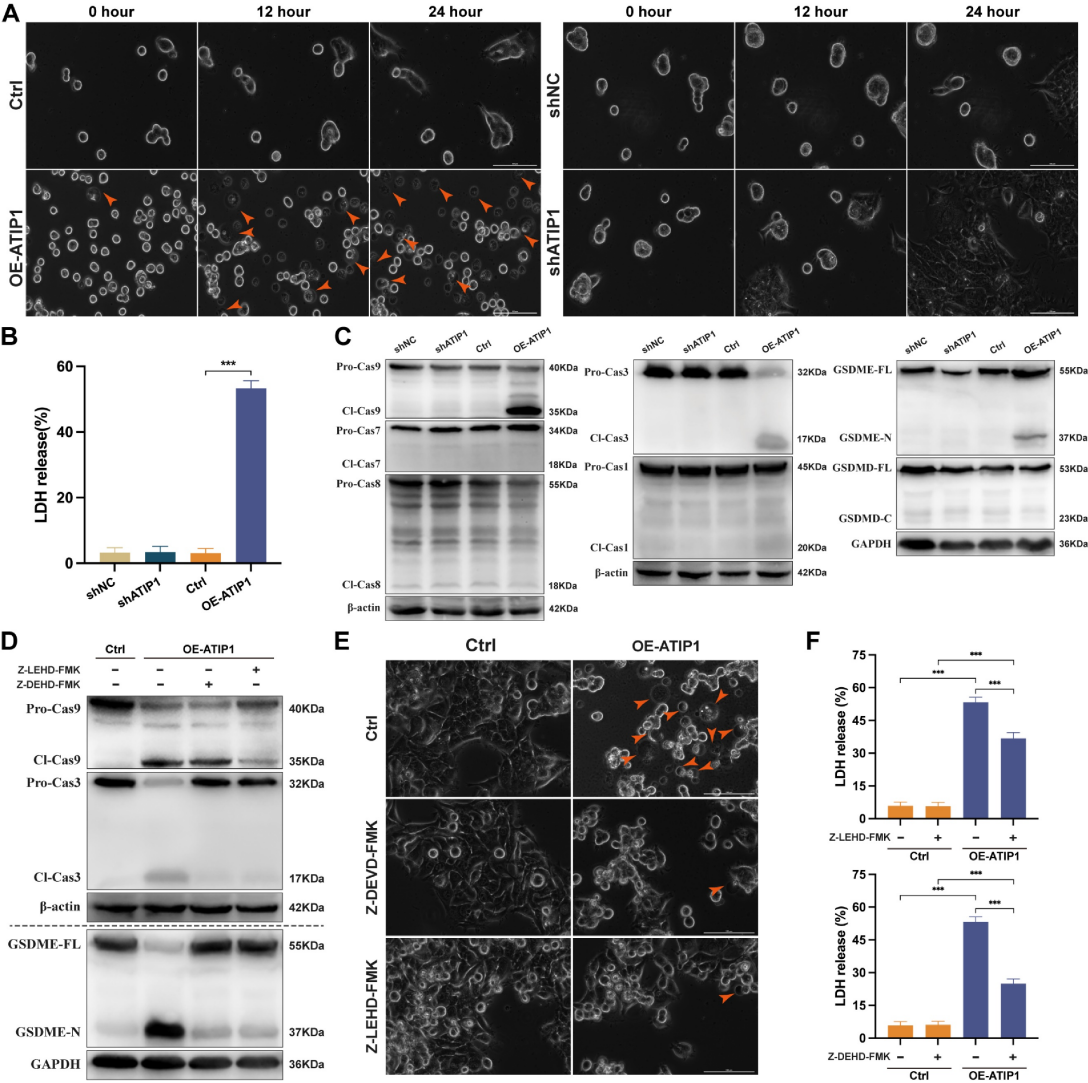
** MTUS1/ATIP1 overexpression induced mitochondrial pyroptosis in HNSCC cells.** (**A-C**) MTUS1/ATIP1-overexpressed or -knockdown HNSCC SCC9 cells were detected by high-content Microscopy imaging, LDH release assay and pyroptotic markers assay. MTUS1/ATIP1 overexpression induced pyroptosis (A), LDH release (B) and GSDME cleavage and Cas-9/-3 cleavage (C). (**D-F**) MTUS1/ATIP1-overexpressed SCC9 cells were treated with Cas-9 inhibitor Z-LEVD-FMK (20 μM) or Cas-3 inhibitor Z-LEHD-FMK (20 μM) for 2 h. Z-LEHD-FMK could lead to the blockade of Cas-9 and -3 cleavage. Z-DEVD-FMK could only lead to the blockade of Cas-3 cleavage. Cas-9/-3 inhibitors blocked MTUS1/ATIP1-induced GSDME cleavage (D), pyroptosis (E) and LDH release (F). Arrowheads indicate the large bubbles emerging from the plasma membrane. Scale bar, 20 μm. GSDME, gasdermin E; GSDMD, gasdermin D; FL, full-length; -N, cleaved N-terminal; -C, cleaved C-terminal; Pro-Cas, pro-caspase; Cl, cleaved. β-actin or GAPDH was used as loading proteins. All data are presented as the mean ± SEM of three independent experiments. ***P < 0.001.

To observe cellular pyroptosis, a high-intensity confocal microscope was employed for cell visualization. More and more HNSCC cells showed as cell swelling and large plasma membrane bubbles in MTUS1/ATIP1-overexpressed group compared with control group (Figure 3A and S3A and Video S3-S4). This phenomenon was not observed in MTUS1/ATIP1-knockdown group and its control group (Figure 3A and S3A). Moreover, LDH concentrations was significant increase in the supernatant of MTUS1/ATIP1-overexpressed group compared with control group, but no differences was detected between MTUS1/ATIP1-knockdown group and its control group (Figure 3B). These alterations suggested the occurrence of pyroptosis in MTUS1/ATIP1-overexpressed HNSCC cells. The pyroptotic markers cleaved caspase-9 (Cl-Cas9), Cl-Cas3 and N-terminal of Gasdermin-E (GSDME-N), but not Cl-Cas1/7/8 or C-terminal of Gasdermin-D (GSDMD-C), were detected in MTUS1/ATIP1-overexpressed HNSCC cells. None of these pyroptotic markers were detected in MTUS1/ATIP1-knockdown cells (Figure 3C and S3B). Moreover, the Cas-9 inhibitor Z-LEHD-FMK and the Cas-3 inhibitor Z-DEVD-FMK inhibited GSDME cleavage (Figure 3D and S3C-D), the pyroptotic phenotype (Figure 3E) and LDH release (Figure 3F). These data showed that MTUS1/ATIP1 activated GSDME-dependent pyroptosis in a Cas-9/-3-dependent manner.

**Figure 4:**
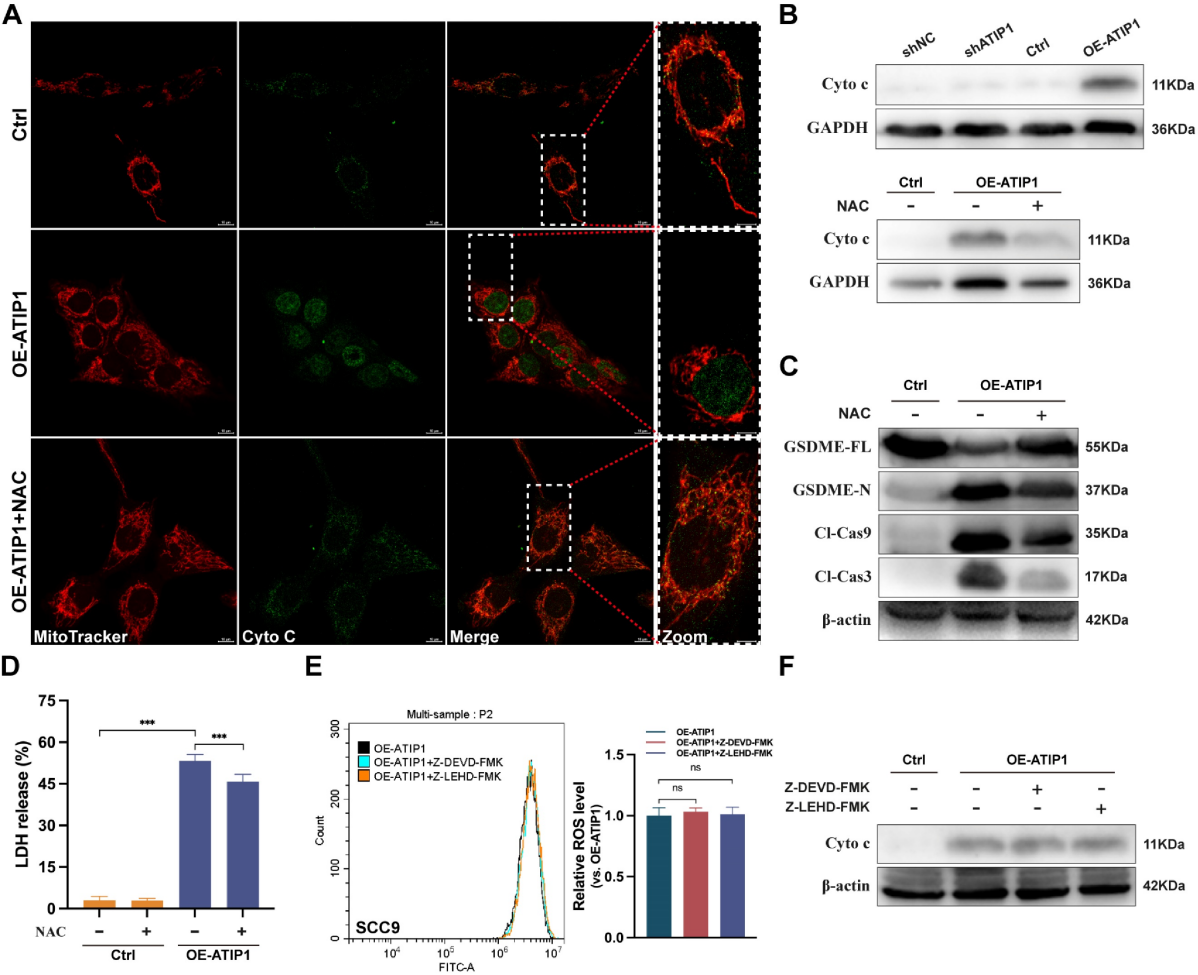
** MTUS1/ATIP1 induced ROS-dependent mitochondrial pyroptosis in HNSCC cells.** MTUS1/ATIP1-overexpressed HNSCC SCC9 cells treated with or without ROS inhibitors NAC (5 mM) for 2h were used to detect Cyto c release, Cas-9/-3 cleavage, pyroptotic features (including LDH release and GSDME cleavage) and ROS level. (**A-B**) MTUS1/ATIP1-induced Cyto c release from mitochondria to cytosol was abolished by NAC in SCC9 cells. Cyto c release was detected by confocal microscopy (A) or western blotting in the cytosol fraction (B). Scale bar, 20 μm. (**C-D**) Cleavage of Cas-9 and -3 (C) and LDH release (D) induced by MTUS1/ATIP1 overexpression can be abolished by NAC in SCC9 cells. (**E-F**) Cas-9 and -3 inhibitor had no effect on the MTUS1/ATIP1-overexpressed-induced ROS elevation (E) and Cyto c release (F) in SCC9 cells. Cyto c, cytochrome c; Cl, cleaved; GSDME, gasdermin E; FL, full-length; -N, cleaved N-terminal. β-actin or GAPDH was used as loading proteins. All data are presented as the mean ± SEM of three independent experiments. ns, not statistically significant (*P*≥ 0.05), ***P < 0.001.

To further investigate the role of ROS in pyroptosis, the ROS inhibitor N-acetyl-L-cysteine (NAC) was incubated with MTUS1/ATIP1-overexpressed or knockdown HNSCC cells. We found that MTUS1/ATIP1 overexpression induced cytochrome c release from mitochondria to the cytosol, which can be abolished by NAC, while MTUS1/ATIP1 knockdown had no effect on cytochrome c release (Figure 4A-B and S4A). NAC also blocked the MTUS1/ATIP1-induced cleavage of Cas-9/-3 and GSDME and LDH release (Figure 4C-D and S4B). However, neither Z-LEHD-FMK nor Z-DEVD-FMK increased ROS or cytochrome c release in MTUS1/ATIP1-overexpressed HNSCC cells (Figure 4E-F and S4C-D). Thus, ROS signaling may act as an upstream factor promoting cytochrome c release, thereby inducing mitochondrial pyroptosis via the cleavage of GSDME by Cas-9/-3 in MTUS1/ATIP1-overexpressed HNSCC cells.

### MTUS1/ATIP1 interacted with MFN2 and led to ROS-induced mitochondrial pyroptosis

**Figure 5:**
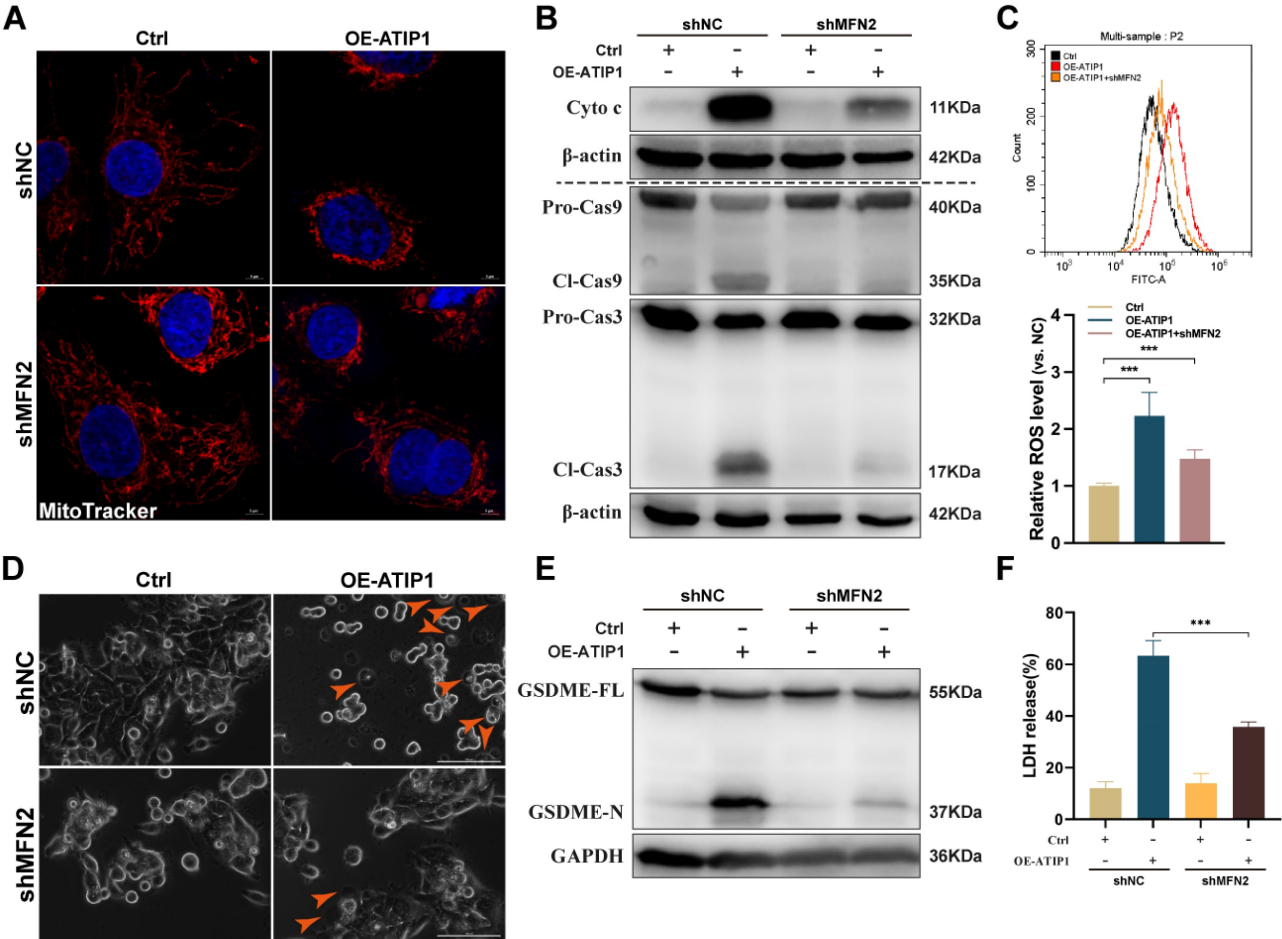
** MTUS1/ATIP1 interacted with MFN2 and led to ROS-induced mitochondrial pyroptosis in HNSCC cells.** (**A**) MFN2 knockdown blocked MTUS1/ATIP1 induced mitochondrial collapse. Scale bar, 20 μm. (**B-C**) MFN2 knockdown inhibited MTUS1/ATIP1-induced Cyto c release, Cas-9 and -3 cleavage (B) and ROS elevation (C) in HNSCC SCC9 cells. (**D-F**) MFN2 knockdown inhibited MTUS1/ATIP1-induced pyroptosis (D), GSDME cleavage (E) and LDH release (F) in HNSCC SCC9 cells. Scale bar, 100 μm. Cyto c, cytochrome c; Cl, cleaved; GSDME, gasdermin E; FL, full-length; -N, cleaved N-terminal. β-actin or GAPDH was used as loading proteins. All data are presented as the mean ± SEM of three independent experiments. ***P < 0.001.

To further assess the contribution of MFN2 to MTUS1/ATIP1-induced mitochondrial pyroptosis, MTUS1/ATIP1-overexpressed HNSCC cells were coinfected with MFN2 shRNA (Figure S5A). As shown in Figure 5A-C and S5B-C, MFN2 knockdown inhibited MTUS1/ATIP1-induced mitochondrial collapse, cytochrome c release, Cas-9/-3 cleavage and ROS produce. Moreover, the pyroptotic phenotype, GSDME cleavage and LDH release were decreased in MTUS1/ATIP1-overexpressed HNSCC cells when coinfected with MFN2 shRNA (Figure 5D-F and S5D-E). MFN2 knockdown had no effect mitochondrial collapse, cytochrome c release, Cas-9/-3 cleavage, pyroptosis induction, GSDME cleavage or LDH release in HNSCC cells (Figure 5 and S5).

**Figure 6:**
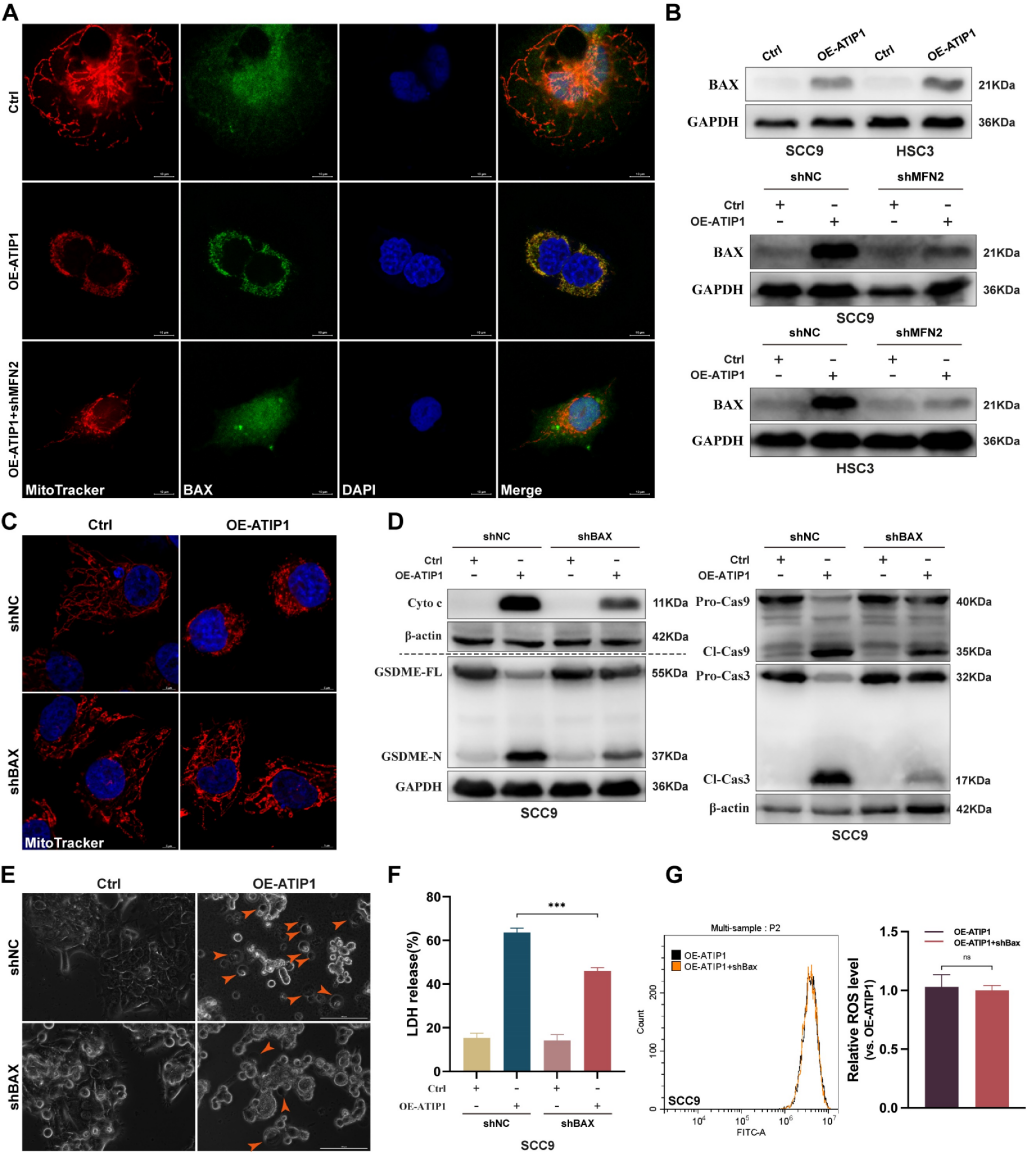
** MFN2 recruited BAX to mitochondria and induced pyroptosis in MTUS1/ATIP1 overexpression HNSCC cells.** (**A-B**) MFN2 knockdown blocked the translocation of BAX to mitochondria by confocal microscopy (A) and western blotting (B) in MTUS1/ATIP1 overexpression HNSCC cells. Scale bar, 10 μm. (**C-F**) BAX knockdown inhibited the MTUS1/ATIP1-induced mitochondrial collapse (C), Cyto c release, Cas-9 and Cas-3 cleavage, GSDME cleavage (D), pyroptosis (E) and the LDH release (F) in MTUS1/ATIP1 overexpression SCC9 cells. Scale bar, 100 μm (C). Scale bar, 5 μm (E). (**G**) BAX had no effect on the MTUS1/ATIP1-induced ROS elevation in MTUS1/ATIP1 overexpression SCC9 cells. Cyto c, cytochrome c; Cl, cleaved; GSDME, gasdermin E; FL, full-length; -N, cleaved N-terminal. β-actin or GAPDH was used as loading proteins. All data are presented as the mean ± SEM of three independent experiments. ns, not statistically significant (*P*≥ 0.05), ***P < 0.001.

To further clarify the mechanism of MFN2 in MTUS1/ATIP1-induced pyroptosis, pro-apoptotic member BAX was investigated. As shown in Figure 6A, BAX was sparsely distributed in the cytosol in control HNSCC cells but overlapped with mitochondria in MTUS1/ATIP1-overexpressed cells; MFN2 knockdown decreased the BAX overlapped with mitochondria in MTUS1/ATIP1-overexpressed cells. Mitochondrial extraction from HNSCC cells showed that mitochondrial BAX was increased in MTUS1/ATIP1-overexpressed HNSCC cells but decreased after cotransfection with shMFN2 (Figure 6B). These results indicated MFN2 recruited mitochondrial localization of BAX in MTUS1/ATIP1-overexpressed HNSCC cells.

To further clarify the role of BAX in the MTUS1/ATIP1-mediated pyroptosis pathway, MTUS1/ATIP1-overexpressed HNSCC cells were coinfected with BAX shRNA (Figure S6A). We found that BAX knockdown not only decreased MTUS1/ATIP1-induced mitochondrial collapse, cytochrome c release, and Cas-9/-3 and GSDME cleavage (Figure 6C-D and S6B-C), but also suppressed the pyroptotic phenotype (Figure 6E) and LDH release (Figure 6F and S6D) in HNSCC cells. However, BAX knockdown did not affect intracellular ROS levels in MTUS1/ATIP1-overexpressed HNSCC cells (Figure 6G and S6E). Moreover, BAX knockdown had no effect on mitochondrial collapse, cytochrome c release, Cas-9/-3 and GSDME cleavage, the pyroptotic phenotype or LDH release in HNSCC cells (Figure 6 and S6). Thus, MTUS1/ATIP1 interacted with MFN2, increased ROS and then recruited BAX translocation to the mitochondria to trigger mitochondria-mediated GSDME-dependent pyroptosis.

### MTUS1/ATIP1 exerted anticancer effects in HNSCC

**Figure 7:**
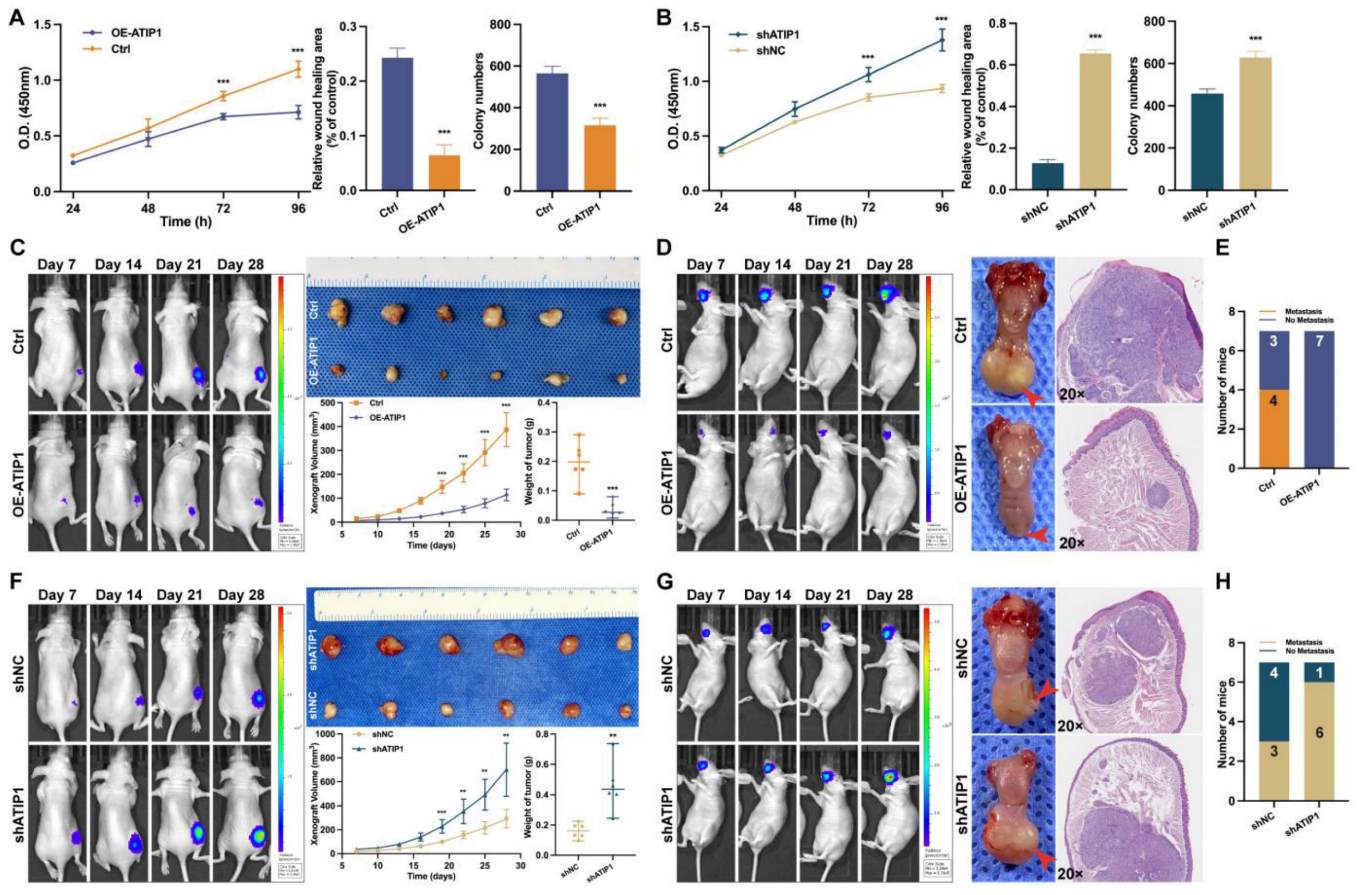
** MTUS1/ATIP1 exerted anticancer effects on HNSCC *in vitro* and *in vivo*.** (**A**) MTUS1/ATIP1 overexpression inhibited the cell proliferation, migration and colony formation abilities in SCC9 cells. (**B**) MTUS1/ATIP1 knockdown enhanced the cell proliferation, migration and colony formation abilities in SCC9 cells. (**C and F**) Representative fluorescence intensity images of tumor-bearing mice, photograph of xenografts and its growth curves, and tumor weights in subcutaneous xenograft of MTUS1/ATIP1 overexpressed (C) or knockdowned mice (F). (**D and G**) Representative fluorescence intensity images of tumor-bearing mice, images of tongue lesions and H&E-staining image in in sito tongue tumor of MTUS1/ATIP1 overexpressed (D) or knockdowned mice (G) . Arrow indicates the location of palpable tongue tumor. (**E and H**) Representative the proportion of mice with lymph node metastases in lymph node metastasis model of MTUS1/ATIP1 overexpressed (E) or knockdowned mice (H). All data are presented as the mean ± SEM of three independent experiments. **P < 0.01; ***P < 0.001.

To investigate the functional role of MTUS1/ATIP1, HNSCC cells were infected with lentivirus containing ATIP1 cDNA or ATIP1 shRNA. As shown in Figure 7A and Figure S7A-B, MTUS1/ATIP1 overexpression in HNSCC cells inhibited cell proliferation, migration and colony formation *in vitro*. However, knockdown of MTUS1/ATIP1 exerted the opposite effects on HNSCC cells (Figure 7B and S8A-B).

*In vivo*, subcutaneous xenografts (xenograft volume and tumor weight) and in situ tongue tumors (tumor size) in MTUS1/ATIP1-overexpressed mice were significantly inhibited compared with those in control mice (Figure 7C-D). The relative fluorescence intensity of subcutaneous xenograft tumors and in situ tongue tumors in the MTUS1/ATIP1 overexpression group was significantly weaker than that in the control group (Figure S7C). In the lymph node metastasis model, MTUS1/ATIP1 overexpression resulted in a significant decrease in lymph node metastasis (Figure 7E). However, the same manipulation led to the opposite results in MTUS1/ATIP1-knockdown mice (Figure 7F-H and Figure S8C).

**Figure 8:**
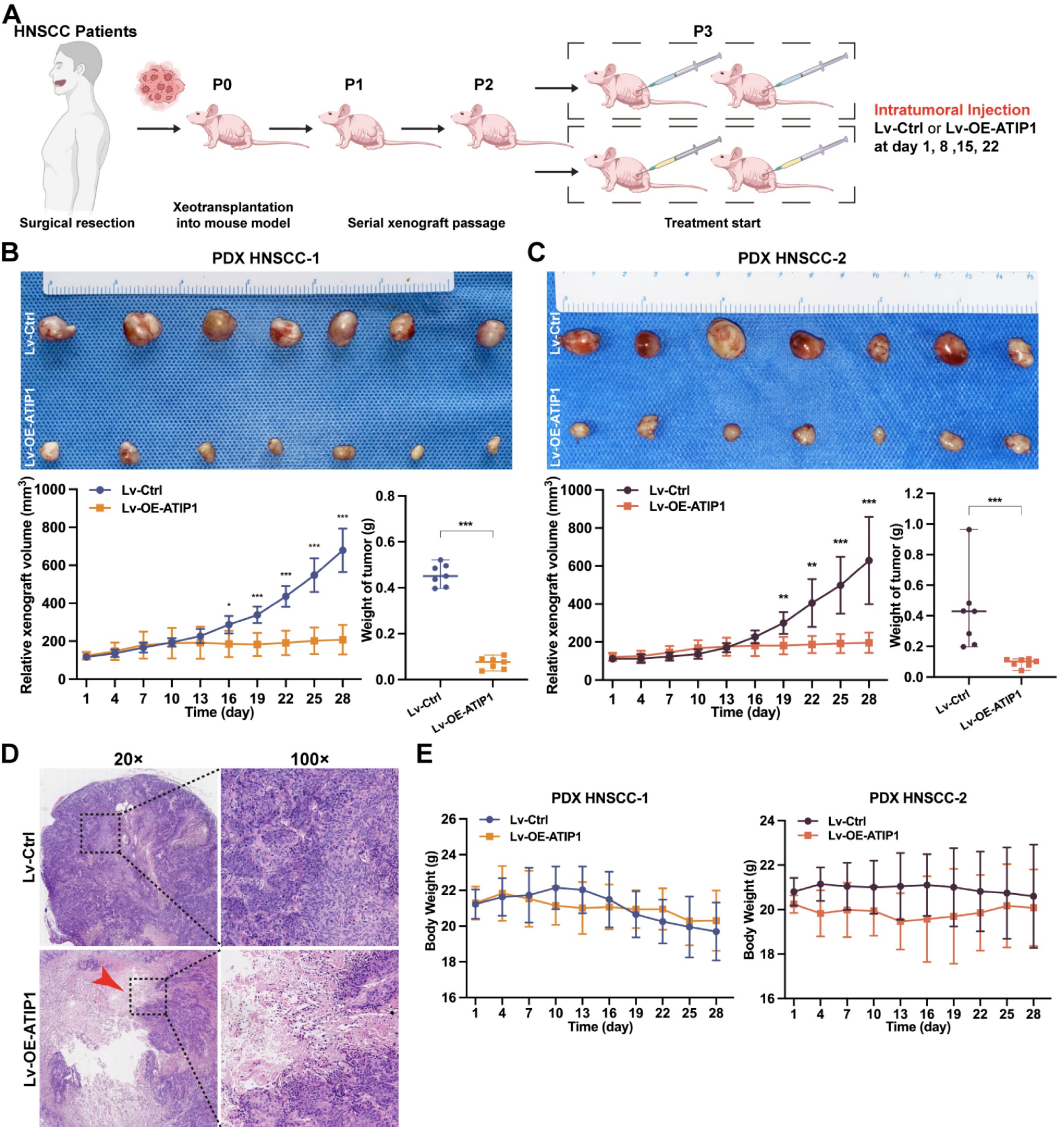
** MTUS1/ATIP1 exerted antitumor effects in HNSCC PDX mouse models.** (**A**) Schema for the establishment of HNSCC PDX models. Mice were treated by intratumoral injection of 10^8^ TU of Lv-OE-ATIP1 or Lv-Ctrl once a week for 4 times. (**B-C**) Representative of xenografts photograph, growth curves and tumor weights in Lv-OE-ATIP1 treated HNSCC PDX models generated from two HNSCC patient samples (PDX HNSCC-1 and PDX HNSCC-2). (**D**) H&E staining showed a large area of tumour tissue necrosis (Arrow) in Lv-OE-ATIP1 treated group. (**E**) No significant differences in body weight was found between Lv-Ctrl and Lv-OE-ATIP1 groups. Data are presented as the mean ± SD (n = 7). *P < 0.05; **P < 0.01; ***P < 0.001.

To further examine the *in vivo* antitumor efficacy by targeting MTUS1/ATIP1, HNSCC PDX models were treated with the MTUS1/ATIP1-overexpressed lentivirus (Lv-OE-ATIP1) or empty lentivirus (Lv-Ctrl) by intratumoral multiple-point injection (Figure 8A). Compared with those in the control group, tumor sizes in the treatment groups began to significantly decrease after 16 days of treatment with Lv-OE-ATIP1. Tumor growth inhibition (TGI) in the intratumoral injection groups was 85.1% and 85.7% after treatment for 28 days (Figure 8B-C). A large area of tumor tissue necrosis was observed in Lv-OE-ATIP1-treated group (Figure 8D), and no significant differences in mouse weight were found between the groups (Figure 8E). Moreover, the HNSCC PDX models, treated via tail vein injection of Lv-OE-ATIP1, had a similar anticancer effect. The TGI rates were 65.1% and 81.3% (Figure S9. No serious side effects, such as mucositis, weakness, or diarrhea, were observed in any of the mice.

## Discussion

MTUS1 expression is downregulated in several types of malignancies, such as breast cancer 37, colorectal carcinoma 38 and gastric cancer 39. ATIP1, which is one of the major products of the MTUS1 gene, plays a pivotal role in the carcinogenesis of several malignancies 40. Our previous study verified that HNSCC cells showed notably reduced ATIP1 expression 13,14. In this study, we further demonstrated that MTUS1/ATIP1 overexpression prevented HNSCC cell self-renewal, proliferation and migration *in vitro*. Subcutaneous and tongue xenograft tumors, lymph node metastasis models and PDX models in BALB/c mice were used to further verify that MTUS1/ATIP1 inhibited HNSCC tumorigenesis and lymph node metastasis. This *in vivo* report was the first to support this notion and revealed that MTUS1/ATIP1 showed promising antitumor activity, as indicated by a tumor inhibition rate up to 85.7%. Thus, targeting MTUS1/ATIP1 may be an effective treatment for HNSCC.

Microtubule-associated tumor suppressor gene (MTUS1), also known as microtubule-associated scaffold protein 1, encodes a family of angiotensin II (AT2) receptor-interacting proteins (ATIP). As a mitochondrial tumor suppressor gene, MTUS1/ATIP1 had not been verified to localize at mitochondria. Rodrigues-Ferreira *et al.* and Molina *et al.* reported that MTUS1/ATIP3 was localized with the centrosome and along the microtubule lattice in interphase and decorated the mitotic spindle and spindle poles during mitosis 26,41. Rodrigues-Ferreira *et al.* also reported that ATIP4 was predicted to localize with the plasma membrane and might be involved in AT2 receptor signaling 27. However, research on the subcellular localization of MTUS1/ATIP1, especially its localization and role in tumor cells, is relatively limited. Nouet *et al.* reported that MTUS1/ATIP1 was localized at mitochondria in Chinese hamster ovary cells 42. MTUS1/ATIP1 was also suggested to be a Golgi and nuclear protein in COS-7 cells 43. In the present study, we identified for the first time that MTUS1/ATIP1 was localized in the outer mitochondrial membrane in HNSCC cells.

MOM proteins mediate mitochondrial fusion, fission and motility 44 and play an important role in maintaining mitochondrial outer membrane permeabilization (MOMP), which influences mitochondrial function and cellular homeostasis 45. MFN2 is a critical component of MOM protein 46 and has been implicated in additional mitochondrial functions, including mitochondrial metabolism 47, mitochondrial apoptosis and energy supply 48. Wang *et al.* found that MFN2 overexpression changed the balance between mitochondrial fusion and fission and promoted intracellular ROS production, resulting in apoptosis in hepatocellular carcinoma cells 49. In this study, we found that MTUS1/ATIP1 interacted with MFN2 protein. MTUS1/ATIP1 overexpression, accompanied by high MFN2 expression, increased the accumulation of perinuclear and collapsed mitochondria, impaired mitochondrial motility and decreased directed motion. In this study, we also found MTUS1/ATIP1 increased ROS produce and inhibited mitochondrial metabolism. Our data indicated that MTUS1/ATIP1 colocalized with MFN2 played an important role in maintaining normal architecture and function and the subcellular distribution of mitochondria in HNSCC cells.

**Figure 9:**
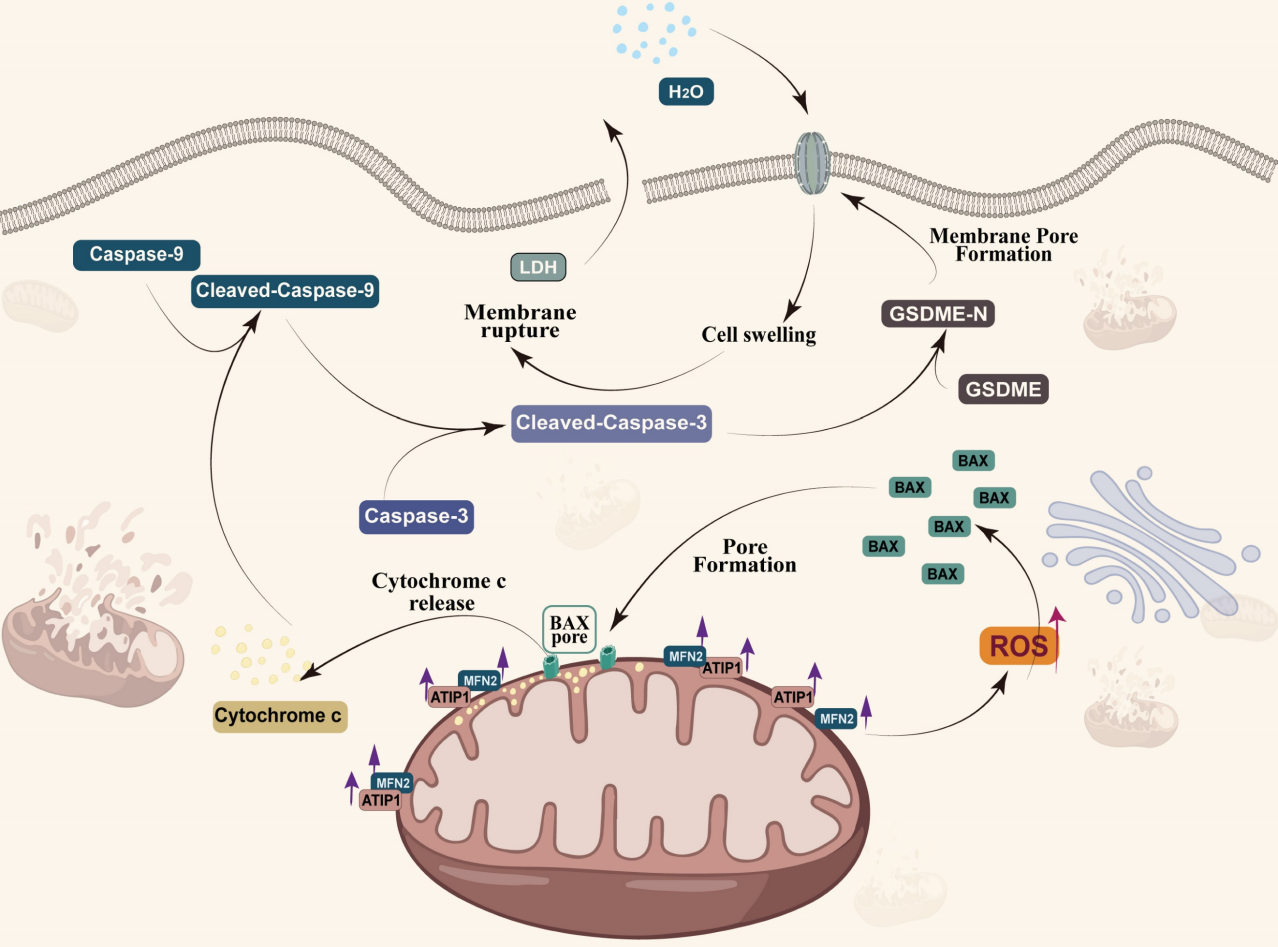
MTUS1/ATIP1 is localized at the mitochondrial outer membrane and interacts with MFN2, produces ROS and induces BAX translocation to mitochondria, which facilitats cytochrome c release to cytosol. Once released, cytochrome c activates caspase-9, which then activates caspase-3 and cleaves GSDME, and eventually triggers pyroptotic cell death.

Pyroptosis played a complex role in the initial and development of cancer 22,50. So *et al.* found that pyroptotic death signaling was inhibited in human papillomavirus (HPV) infected cancer cells, which resulted in poor clinical prognosis of cervical cancer 51. Wang *et al.* also reported that pyroptosis-induced inflammation within the tumour microenvironment can induce antitumour immunity 21. At least two different kinds of signaling pathways have been reported to initiate pyroptosis 52. Traditionally, it is believed that pyroptosis is a pathogen-induced pathway, in which bacteria and microbial infections, activates caspase-1/4/5/11 to cleave gasdermin D (GSDMD) to generate the N-terminal and C-terminal fragments 53,54. Recent studies have demonstrated that pyroptosis can also be activated by the caspase-3-GSDME pathway 25,55,56. Zhang *et al.* found that GSDME mediated pyroptosis downstream of the ROS/JNK-mitochondrial apoptotic pathway in breast cancer 24. We also found that MTUS1/ATIP1 could induce HNSCC cell pyroptosis. Overexpression of MTUS1/ATIP1 recruited BAX translocation to the mitochondria, released cytochrome c to cytosol, then cleaved Cas-9/-3 and GSDME and induced pyroptosis (Figure 9). These findings also verified by the previous research of Yu *et al.* in colorectal cancer 23.

Recent studies had proved the interplay between pyroptosis and apoptosis, and pyroptosis can occur as secondary necrosis after apoptosis in certain cells 56. Ren *et al.* reported that GSDME can switch apoptosis to pyroptosis in malignant tumor cells 57. Zhang *et al.* also found that the expression level of GSDME determined whether cells underwent pyroptosis or apoptosis 22. However, GSDME is often downregulated or even silenced in cancer cells due to hypermethylation of its promoter 58,59. In this study, ours and data from The Cancer Genome Atlas (TCGA) showed that GSDME was specifically and highly expressed in HNSCC Figure S10A-B). We also found that MTUS1 were significantly downregulated in HNSCC tissues compared with normal tissues (Figure S10C). Moreover, a significant correlation between GSDME and MTUS1 level was found in HNSCC from TCGA (Figure S10D).

So, the High GSDME expression may explain why MTUS1/ATIP1-mediated pyroptosis induction in HNSCC cells. Our previous study had verified that MTUS1/ATIP1 induced apoptosis (9,14, So pyroptosis maybe a secondary necrosis after apoptosis 55,56,60.

## Conclusions

MTUS1/ATIP1 was localized in the outer mitochondrial membrane and interacted with MFN2, influenced the function and metabolism of mitochondria, and induced pyroptotic death in HNSCC cells via the ROS-BAX-caspase-9/-3-GSDME pathway. The antineoplastic effect of MTUS1/ATIP1 was also verified *in vitro* and *in vivo*. Collectively, MTUS1/ATIP1 may provide a novel, promising therapeutic target for the clinical treatment of HNSCC.

## Supplementary Material

Supplementary figures and tables, video legends.

Supplementary video 1.

Supplementary video 2.

Supplementary video 3.

Supplementary video 4.

## Abbreviations

MTUS1: Microtubule-associated tumor suppressor
AT2: angiotensin II
ATIPs: angiotensin II receptor-interacting proteins
ROS: reactive oxygen species
HNSCC: head and neck squamous cell carcinoma
MFN2: Mitofusin-2
MOM: mitochondrial outer membrane
MOMP: mitochondrial outer membrane permeabilization
BCL2: B-cell lymphoma 2
BAX: BCL2-Associated X
CDX: cell-derived xenograft
PDX: patient-derived xenograft
GSDME: Gasdermin-E
GSDME-N: N-terminal of Gasdermin-E
GSDMD: Gasdermin-D
GSDMD-C: C-terminal of Gasdermin-D
LDH: lactate dehydrogenase
NAC: N-acetyl-L-cysteine
Cas9: caspase-9
Cl-Cas9: cleaved caspase-9
Cas3: caspase-3
Cl-Cas3: cleaved caspase-3
Cyto c: cytochrome c
TGI: tumor growth inhibition
HPV: human papillomavirus
TCGA: The Cancer Genome Atlas
P/S: penicillin/streptomycin
FBS: fetal bovine serum
MOI: multiplicity of infection
shRNA: short hairpin RNA
qRT-PCR: quantitative real-time PCR
CCK-8: Cell Counting Kit-8
SPF: specific pathogen-free
PFA: paraformaldehyde
HOK: human oral keratinocytes
