# Supplementary material for: Mitochondrial outer membrane protein MTUS1/ATIP1 exerts antitumor effects through ROS-induced mitochondrial pyroptosis in head and neck squamous cell carcinoma
Source: Int J Biol Sci. 2024 Apr 22;20(7):2576–91. doi: 10.7150/ijbs.94795 (PMC11077360; doi:10.7150/ijbs.94795)
Supplement: Supplementary file 1 — Supplementary figures and tables, video legends. [file ijbsv20p2576s1.pdf]

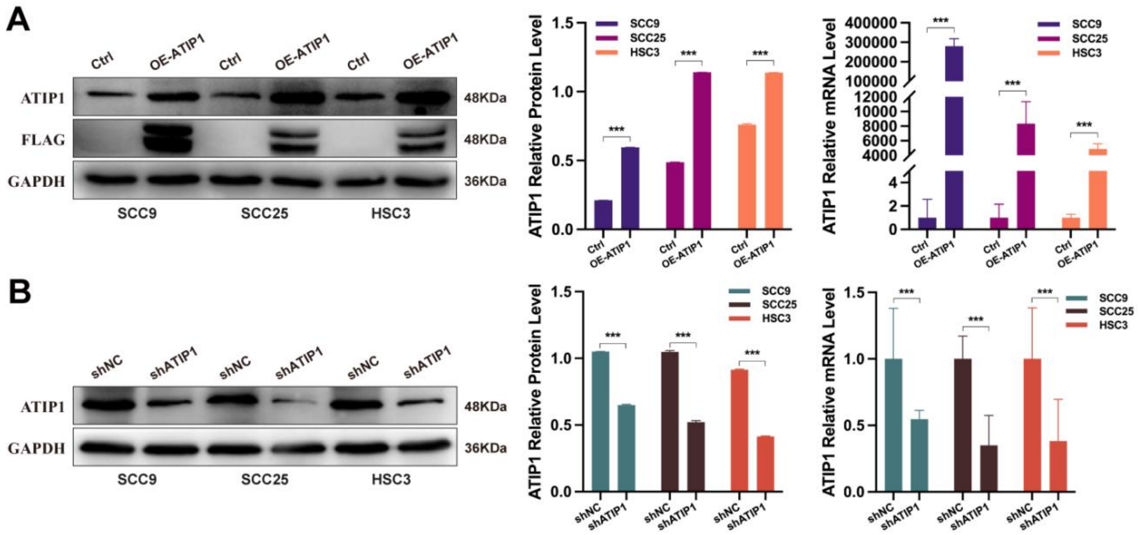

Figure S1. MTUS1/ATIP1 was localized at the outer mitochondrial membrane in HNSCC cells.
(**A**) HNSCC cells infected with MTUS1/ATIP1 cDNA lentivirus were detected by western Blotting and qRT-PCR. (**B**) HNSCC cells were infected with the lentivirus containing shNC or shATIP1 and detected by western Blotting and qRT-PCR. All data are presented as the mean ± SEM of three independent experiments. ****P* < 0.001.

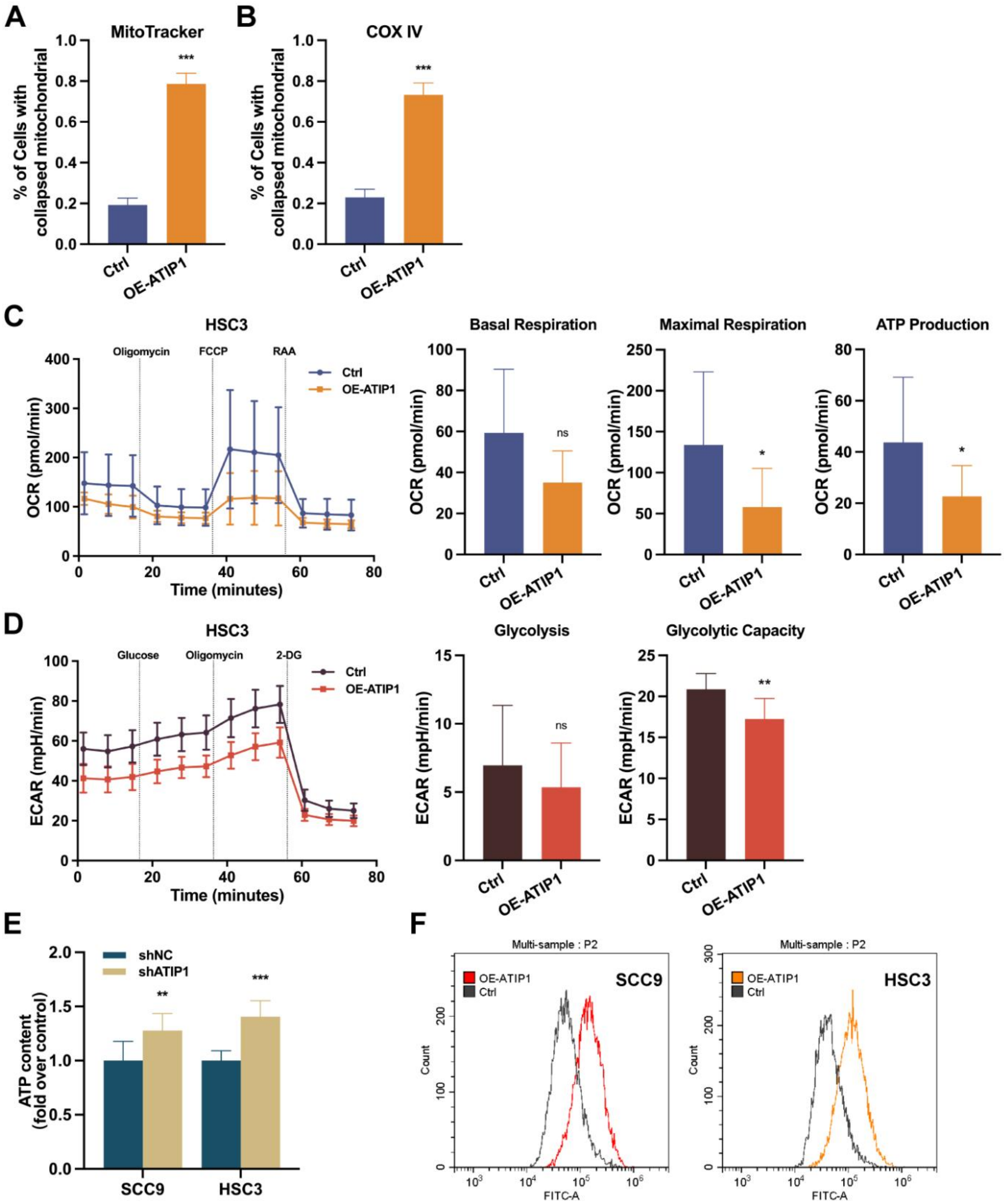

Figure S2. MTUS1/ATIP1 influenced mitochondrial function and metabolism in HNSCC cells. (**A-B**) MitoTracker Red staining or Immunofluorescence staining with COX IV were used to detect the distribution of mitochondria under a confocal microscope in MTUS1/ATIP1-overexpressed or control HNSCC cells. The collapsed mitochondria numbers detected by MitoTracker staining (A) or COX IV staining (B) were quantified respectively. (**C-D**) MTUS1/ATIP1-overexpressed or control HSC3 cells was determined using oxygen consumption rate (OCR) and extracellular acidification rate (ECAR) assays. (**E**) Intracellular ATP levels were measured by a bioluminescence ATP determination assay in MTUS1/ATIP1-knockdwon or control HNSCC cells. (**F**) MTUS1/ATIP1-overexpressed or control HNSCC cells were stained with DCFH-DA and then ROS levels were detected using flow cytometry. All data are presented as the mean ± SEM of three independent experiments. *$P < 0.05$; **$P < 0.01$; ***$P < 0.001$.

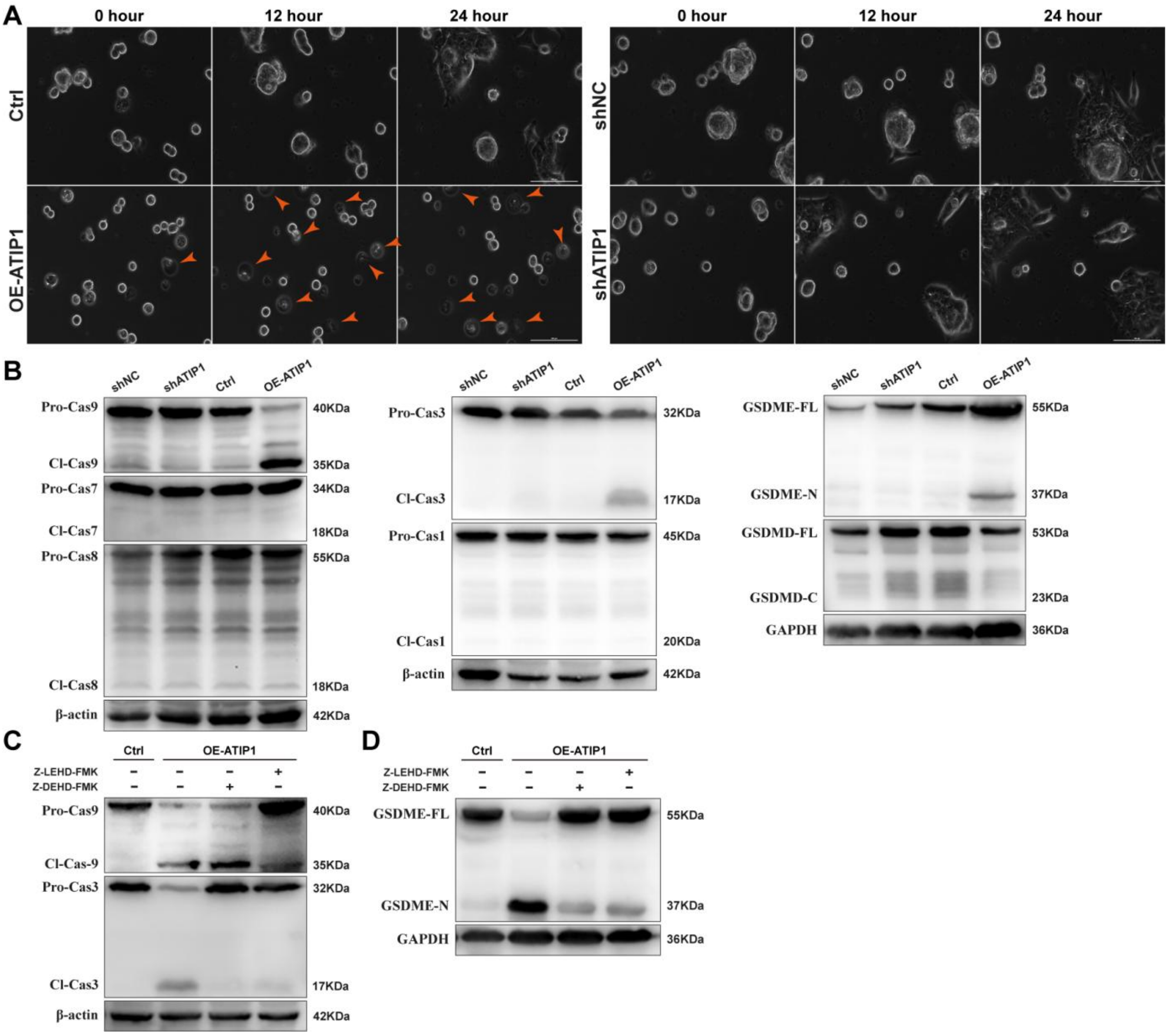

Figure S3. MTUS1/ATIP1 overexpression induced mitochondrial pyroptosis in HSC3 cells.
(**A-B**) MTUS1/ATIP1-overexpressed or -knockdown HSC3 cells were detected by High-content Microscopy imaging and pyroptotic markers assay. MTUS1/ATIP1 overexpression induced pyroptosis (A) and GSDME cleavage and Cas-9/-3 cleavage (B). (**C-D**) MTUS1/ATIP1-overexpressed HSC3 cells were treated with Cas-9 inhibitor Z-LEVD-FMK (20 μM) or Cas-3 inhibitor Z-LEHD-FMK (20 μM) for 2 h. Z-LEHD-FMK could lead to the blockade of Cas-9 and -3 cleavage. Z-DEVD-FMK could only lead to the blockade of Cas-3 cleavage. Cas-9/-3 inhibitors blocked MTUS1/ATIP1-induced GSDME cleavage (C) and pyroptosis (D). Arrowheads indicate the large bubbles emerging from the plasma membrane. Scale bar, 20 μm. GSDME, gasdermin E; GSDMD, gasdermin D; FL, full-length; -N, cleaved N-terminal; -C, cleaved C-terminal; Pro-Cas, pro-caspase; Cl, cleaved. β-actin or GAPDH was used as loading proteins.

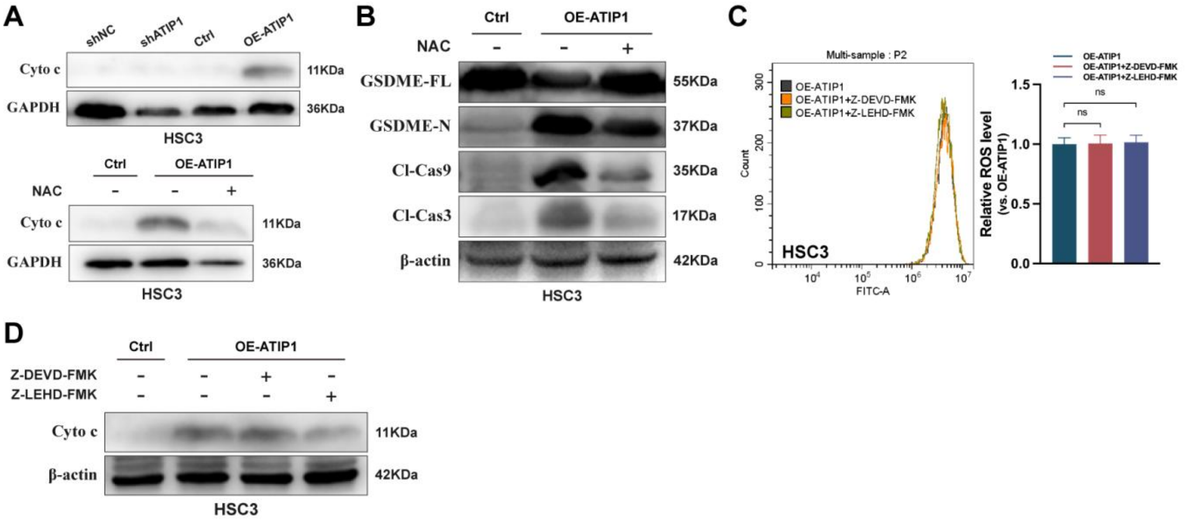

Figure S4. MTUS1/ATIP1 induced ROS-dependent mitochondrial pyroptosis in HSC3 cells. MTUS1/ATIP1-overexpressed HSC3 cells treated with or without ROS inhibitors NAC (5 mM) for 2 h were used to detect Cyto c release, Cas-9/-3 cleavage, pyroptotic features (including LDH release and GSDME cleavage) and ROS level. (**A**) MTUS1/ATIP1 overexpression induced Cyto c release from mitochondria to cytosol. NAC abolished Cyto c release was detected western blotting in the cytosol fraction. (**B**) NAC abolished MTUS1/ATIP1-induced cleavage of Cas-9 and -3 in HSC3 cells. (**C-D**) Cas-9 or -3 inhibitor had no effect on the MTUS1/ATIP1-induced ROS elevation (C) and Cyto c release (D) in HSC3 cells. Cyto c, cytochrome c; GSDME, gasdermin E; FL, full-length; -N, cleaved N-terminal; Pro-Cas, pro-caspase; Cl, cleaved. β-actin or GAPDH was used as loading proteins. All data are presented as the mean ± SEM of three independent experiments. ns, not statistically significant ($P \geq 0.05$). $^{**}P < 0.01$; $^{***}P < 0.001$.

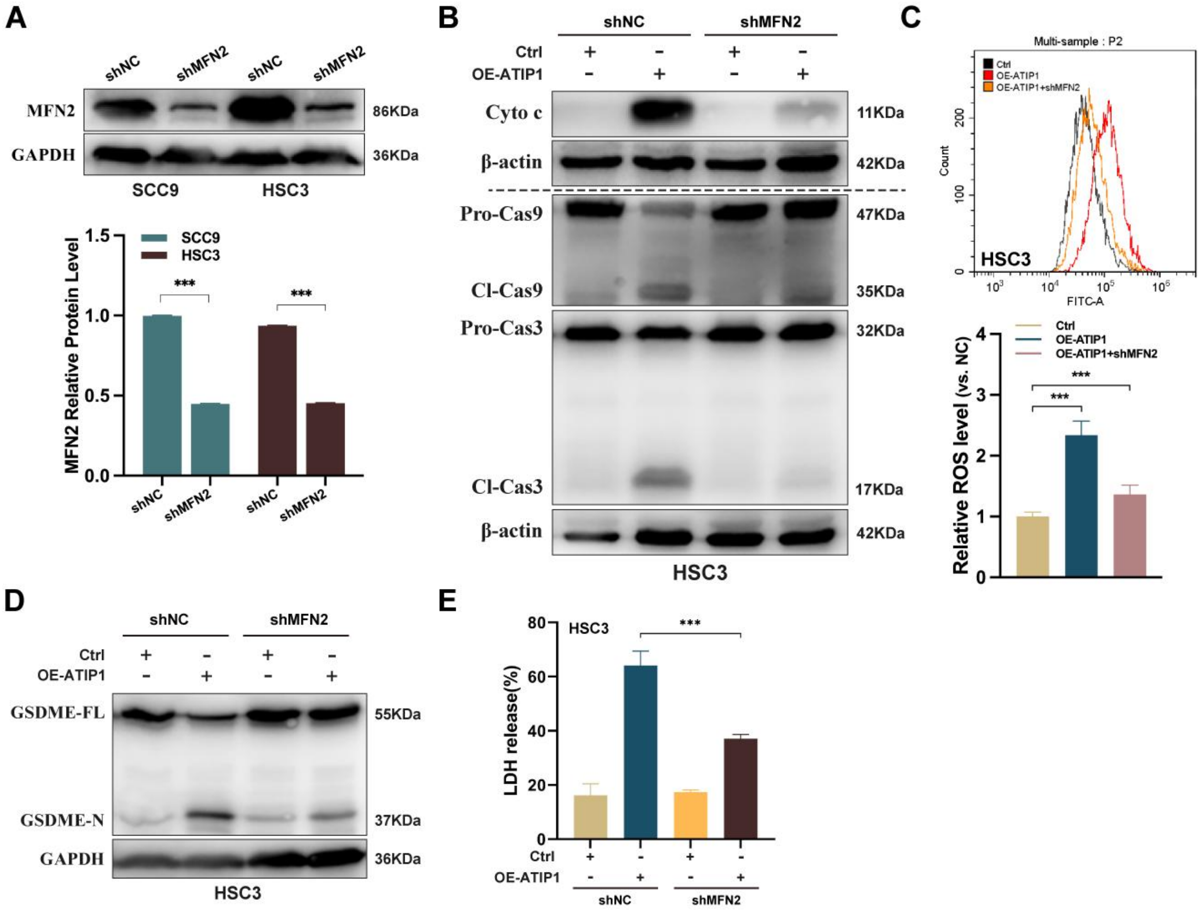

Figure S5. MTUS1/ATIP1 interacted with MFN2 and led to ROS-induced mitochondrial pyroptosis in HNSCC cells.

(**A**) MFN2 knockdown in HNSCC cells was verified by western blotting and qRT-PCR. (**B-C**) MFN2 knockdown inhibited MTUS1/ATIP1-induced Cyto c release, Cas-9 and -3 cleavage (B) and ROS elevation (C) in HSC3 cells. (**D-E**) MFN2 knockdown inhibited MTUS1/ATIP1-induced GSDME cleavage (D) and LDH release (E) in HSC3 cells. Cyto c, cytochrome c; Cl, cleaved; GSDME, gasdermin E; FL, full-length; -N, cleaved N-terminal. β-actin or GAPDH was used as loading proteins. All data are presented as the mean ± SEM of three independent experiments. ***$P < 0.001$.

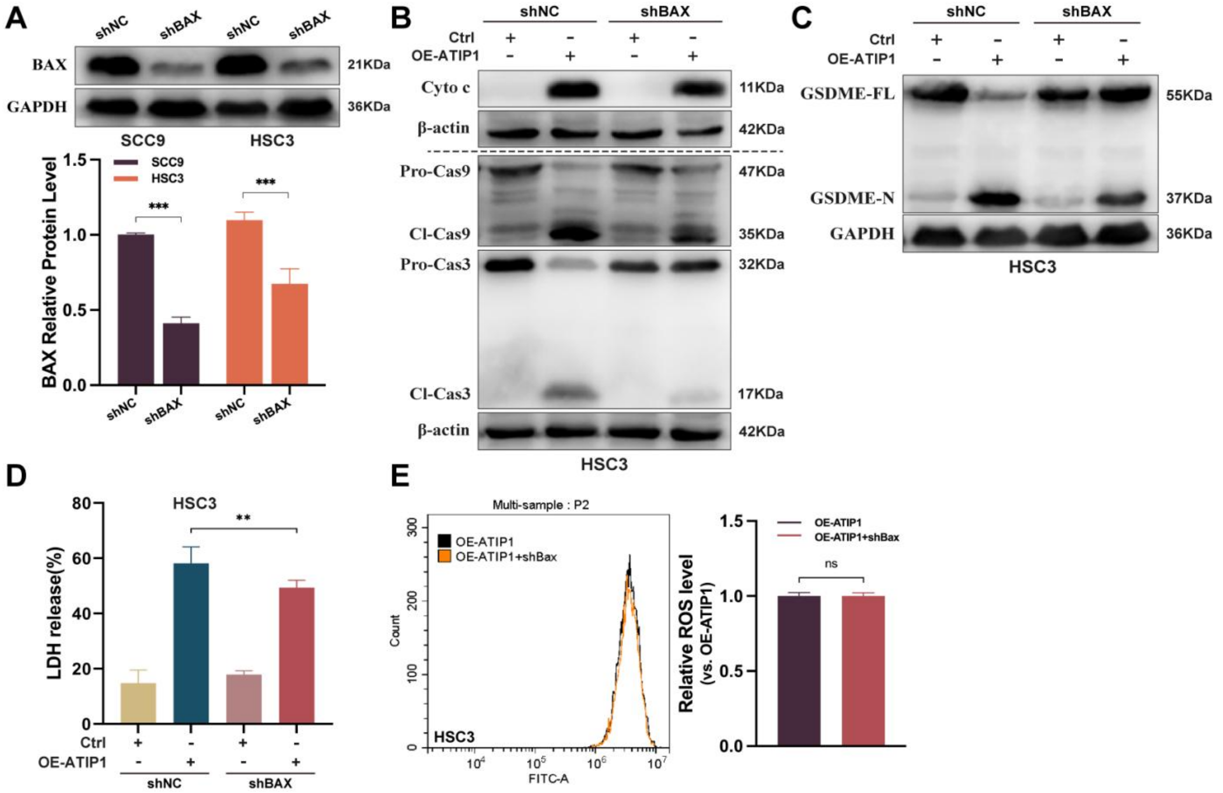

Figure S6. MFN2 recruited BAX to mitochondria and induced pyroptosis in MTUS1/ATIP1 overexpression HNSCC cells.

(**A**) BAX knockdown in HNSCC cells was verified by western blotting. (**B-D**) BAX knockdown inhibited the MTUS1/ATIP1-induced Cyto c release, Cas-9 and Cas-3 cleavage (B), GSDME cleavage (C) and the LDH release (D) in MTUS1/ATIP1 overexpression HSC3 cells. (**E**) BAX had no effect on the MTUS1/ATIP1-induced ROS elevation in MTUS1/ATIP1 overexpression HSC3 cells. Cyto c, cytochrome c; Cl, cleaved; GSDME, gasdermin E; FL, full-length; -N, cleaved N-terminal. β-actin or GAPDH was used as loading proteins. All data are presented as the mean ± SEM of three independent experiments. ns, not statistically significant ($P \geq 0.05$), $**P < 0.01$; $***P < 0.001$.

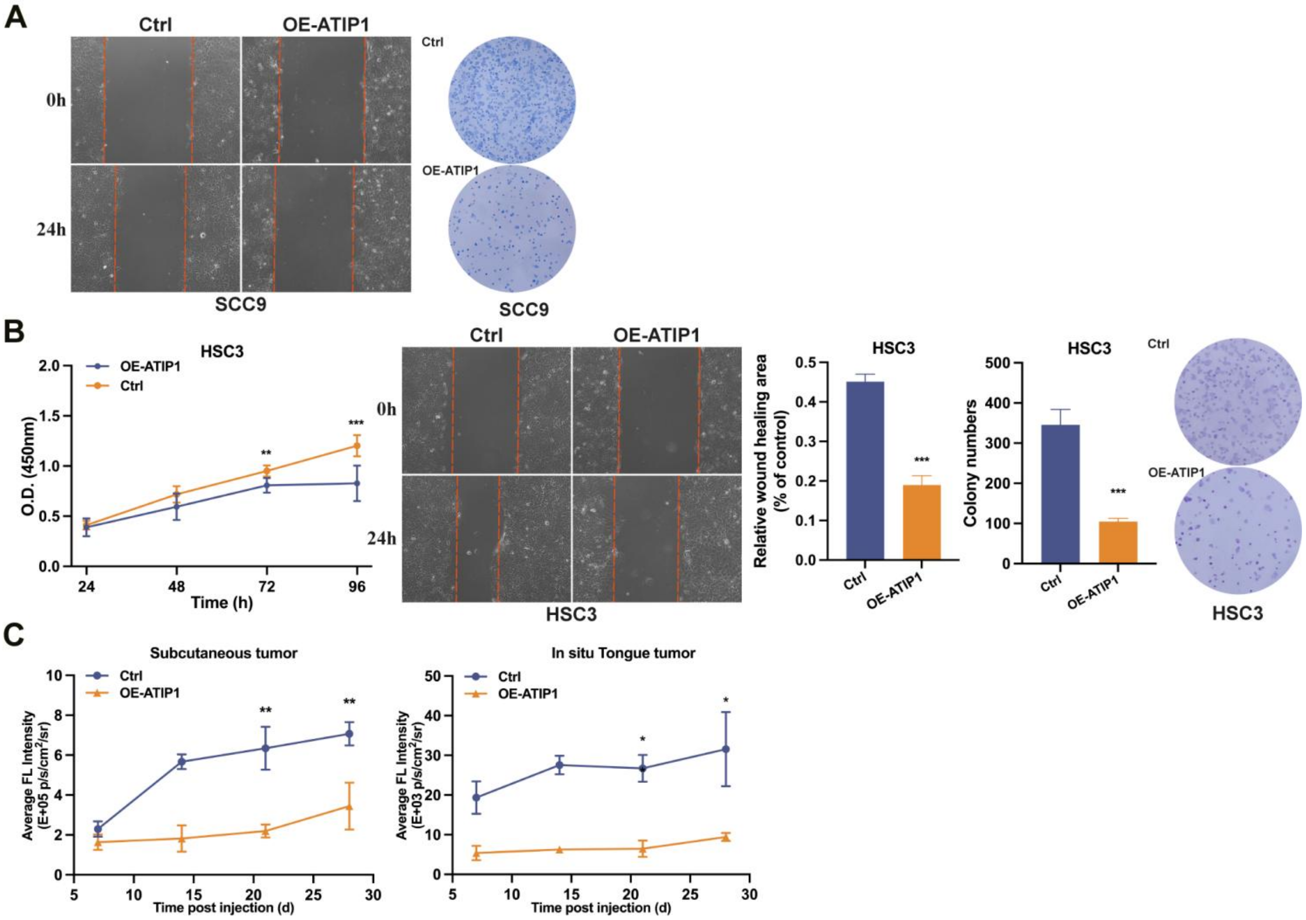

Figure S7. MTUS1/ATIP1 exerted anticancer effects on HNSCC *in vitro* and *in vivo.*
(**A**) MTUS1/ATIP1 overexpression inhibited migration and colony formation abilities in HNSCC cells. (**B**) MTUS1/ATIP1 overexpression inhibited cell proliferation, migration and colony formation abilities in HSC3 cells. (**C**) Quantification of fluorescence intensities at tumor sites in subcutaneous xenograft or in sito tongue tumor in MTUS1/ATIP1 overexpressed mice. FL, fluorescence intensity. All data are presented as the mean ± SEM of three independent experiments. **P* < 0.05; ***P* < 0.01; ****P* <0.001.

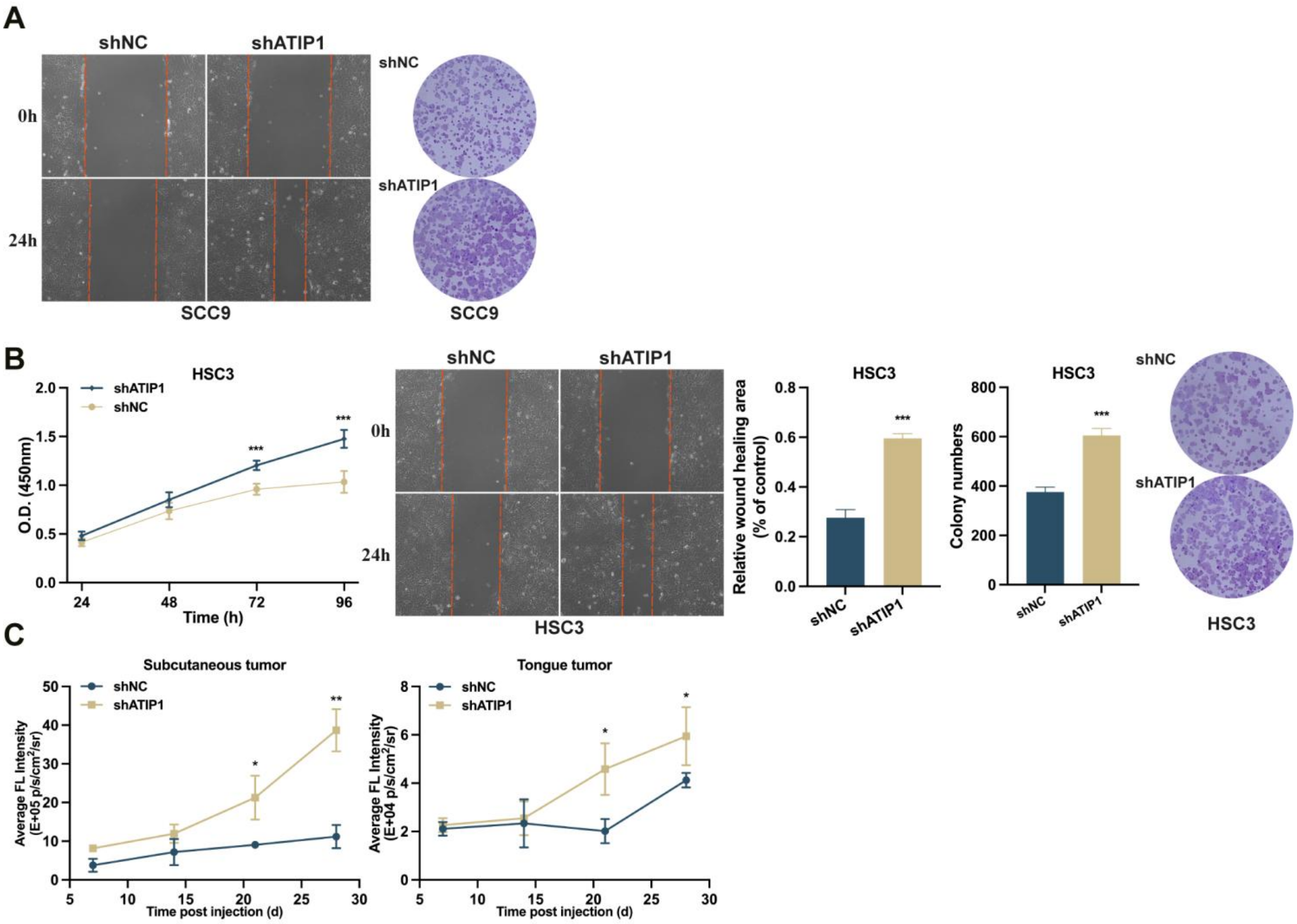

Figure S8. MTUS1/ATIP1 knockdown enhanced the proliferation, migration and growth in HNSCC *in vitro* and *in vivo*.

(**A**) MTUS1/ATIP1 knockdown enhanced migration and colony formation abilities in SCC9 cells. (**B**) MTUS1/ATIP1 knockdown enhanced cell proliferation, migration and colony formation abilities in HSC3 cells. (**C**) Quantification of fluorescence intensities at tumor sites in subcutaneous xenograft or in sito tongue tumor in MTUS1/ATIP1 knockdowned mice. FL, fluorescence intensity. All data are presented as the mean ± SEM of three independent experiments. *P<0.05; **P<0.01; ***P<0.001.

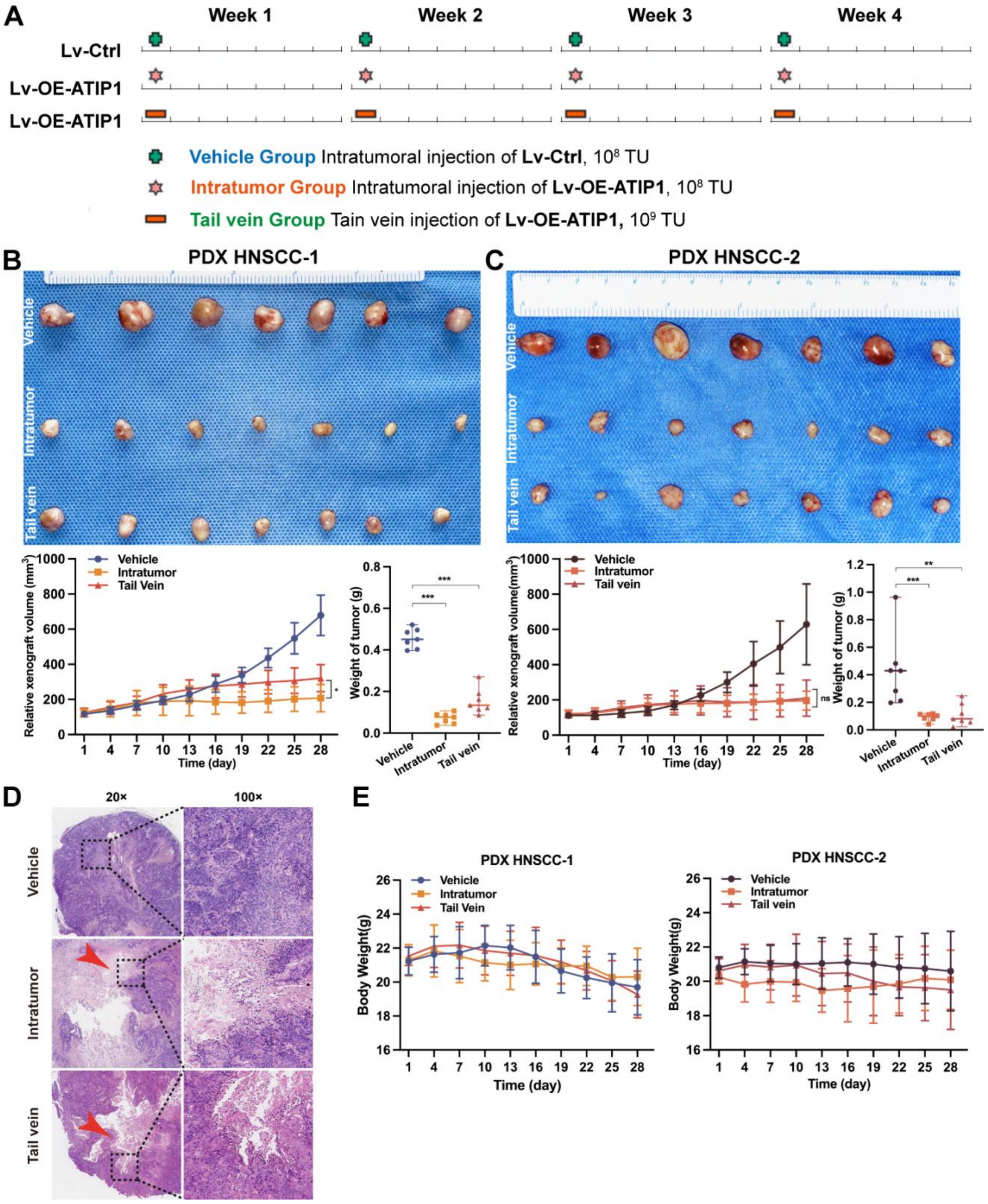

Figure S9. MTUS1/ATIP1 exerted antitumor effects in HNSCC PDX mouse models.
(**A**) Schema of HNSCC PDX animal experiments. (**B-C**) Representative of xenografts photograph, growth curves and tumor weights in Lv-OE-ATIP1 treated HNSCC PDX models generated from two HNSCC patient samples (PDX HNSCC-1 and PDX HNSCC-2). (**D**) H&E staining showed a large area of tumour tissue necrosis (Arrow) in Lv-OE-ATIP1 treated group. (E) No significant differences in body weight was found between Lv-Ctrl and Lv-OE-ATIP1 groups. Data are presented as the mean ± SD (n = 7). **P* < 0.05; ***P* < 0.01; ****P* < 0.001.

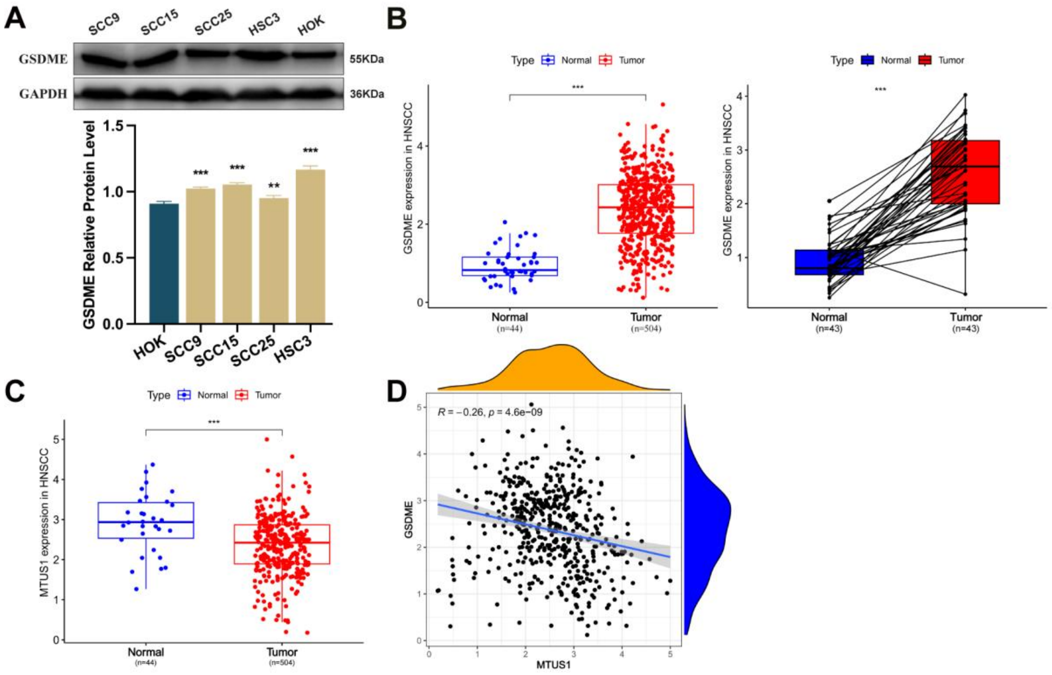

Figure S10. The correlation between MTUS1/ATIP1 and GSDME expression in HNSCC.
(**A**) GSDME expression level in HOK cells and different HNSCC cell lines. (**B**) Transcriptomic analysis of the expression of GSDME between normal tissue and HNSCC tumor samples. Analysis was performed using data from the TCGA with the UALCAN analysis software. (**C**) Transcriptomic analysis of the expression of MTUS1 between normal tissue and HNSCC tumor samples from the TCGA. (**D**) Transcriptomic analysis of the correlation between GSDME and MTUS1 expression level between normal tissue and HNSCC tumor samples in HNSCC from the TCGA. Analysis was performed using data from the TCGA with the UALCAN analysis software. GAPDH was used as loading proteins. All data are presented as the mean ± SEM of three independent experiments. $^{**}P < 0.01$; $^{***}P < 0.001$.

Human oral keratinocytes (HOK) were purchased from ScienCell (Carlsbad, USA) and cultured with RPMI-1640 medium containing 15% fetal bovine serum (FBS) and 1% penicillin/streptomycin (P/S).

Table S1. ShRNA sequences used in the study

**The sequences for MTUS1/ATIP1, MFN2 or BAX shRNA**					
**No.**	**5'**	**stem**	**loop**	**stem**	**3'**
***ATIP1-shRNA1**	Ccgg	cgGGACACTTACATTGAAGAA	CTCGAG	TTCTTCAATGTAAGTGTCCCG	TTTTTg
ATIP1-shRNA2	Ccgg	GGAGGAAGCACTGAAACAACA	CTCGAG	TGTTGTTTCAGTGCTTCCTCC	TTTTTg
ATIP1-shRNA3	Ccgg	GCCTATGAAGCCTCCCTTTCA	CTCGAG	TGAAAGGGAGGCTTCATAGGC	TTTTTg
MFN2-shRNA1	Ccgg	GCAGGTTTACTGCGAGGAAAT	CTCGAG	ATTTCCTCGCAGTAAACCTGC	TTTTTg
MFN2-shRNA2	Ccgg	GTCAAAGGTTACCTATCCAAA	CTCGAG	TTTGGATAGGTAACCTTTGAC	TTTTTg
***MFN2-shRNA3**	Ccgg	GCACTTTGTCACTGCCAAGAA	CTCGAG	TTCTTGGCAGTGACAAAGTGC	TTTTTg
BAX-shRNA1	Ccgg	GCCGGAACTGATCAGAACCAT	CTCGAG	ATGGTTCTGATCAGTTCCGGC	TTTTTg
***BAX-shRNA2**	Ccgg	GCCAGCAAACTGGTGCTCAAG	CTCGAG	CTTGAGCACCAGTTTGCTGGC	TTTTTg
BAX-shRNA3	Ccgg	GCTGTTGGGCTGGATCCAAGA	CTCGAG	TCTTGGATCCAGCCCAACAGC	TTTTTg

*presented as the sequence with the best knockdown effect for ATIP1-shRNA, MFN2-shRNA or BAX-shRNA.

Table S2. The clinical characteristic of HNSCC patients in the PDX models

No.	Age	Sex	Clinical diagnosis	TNM	Pathologic diagnosis
1	46	Female	Left tongue squamous cell carcinoma	$T_3N_2M_0$	Well differentiated
2	68	Male	Right tongue squamous cell carcinoma	$T_1N_0M_0$	Well differentiated

Table S3. The primers for qRT-PCR

Name	Sequence
**ATIP1**	sense: 5'-AGGAGAAGCACTGTTGTTTTCCAC-3'<br>antisense: 5'-GGAGCTGTCTGTGGCTGGATA C-3'
**MFN2**	sense: 5'-CACATGGAGCGTTGTACCAG-3'<br>antisense: 5'-TTGAGCACCTCCTTAGCAGAC-3'
**BAX**	sense: 5'-CCCGAGAGGTCTTTTTCCGAG-3'<br>antisense: 5'-CCAGCCCATGATGGTTCTGAT-3'
**RPLP0**	sense: 5'-GGC GAC CTG GAA GTC CAA CT-3'<br>antisense: 5'-CCA TCA GCA CCA CAG CCT TC-3'
**GAPDH**	sense: 5'-ACAACTTTGGTATCGTGGAAGG-3'<br>antisense: 5'-GCCATCACGCCACAGTTTC-3'

Table S4. Monoclonal or polyclonal antibodies used for western blotting

Antibody	Host	Source	Catalog number
**ATIP1 (N-terminal region)**	Rabbit	Aviva Systems Biology	ARP44418_T100
**MTUS1**	Rabbit	CST	#13436-1-AP
**COX IV**	Mouse	Abcam	ab33985
**Caspase-1**	Rabbit	Abcam	ab207802
**Caspase-3**	Rabbit	Abcam	ab184787
**Caspase-7**	Rabbit	Abcam	ab255818
**Caspase-8**	Rabbit	Abcam	ab32397
**Caspase-9**	Rabbit	CST	#9502
**GSDMD**	Rabbit	Abcam	ab210070
**GSDME**	Rabbit	Abcam	ab215191
**BAX**	Rabbit	Proteintech	50599-2-Ig
**GM130**	Rabbit	Abcam	ab52649
**MFN1/2**	Mouse	Abcam	ab57602
**MFN2**	Rabbit	Abcam	ab124773
**TIMM23**	Rabbit	Abcam	ab230253
**TOMM20**	Mouse	Abcam	ab56783
**Cytochrome C**	Rabbit	Abcam	ab133504
**DYKDDDDK tag**	Rabbit	CST	#14793S
**β-Actin**	Rabbit	CST	#4970
**GAPDH**	Rabbit	CST	#2118

Table S5. Monoclonal or polyclonal antibodies used for immunofluorescence staining

Antibody	Host	Source	Catalog number
**ATIP1 (N-terminal region)**	Rabbit	Aviva Systems Biology	ARP44418_T100
**MTUS1**	Rabbit	CST	#13436-1-AP
**COX IV**	Mouse	Abcam	ab33985
**MFN1/2**	Mouse	Abcam	ab57602
**TOMM20**	Mouse	Abcam	ab56783
**DYKDDDDK tag**	Rabbit	CST	#14793S

Video S1. Mitochondrial real-time motions of Control HNSCC cells under confocal microscope

Video S2. Mitochondrial real-time motions of MTUS1/ATIP1-overexpressed HNSCC cells under confocal microscope

Video S3. HNSCC cells exhibited pyrotosis in MTUS1/ATIP1-overexpressed group (Scale bar, 100 μm)

Video S4. Few pyrotosis was observed in Control HNSCC group (Scale bar, 100 μm)
